# The Limpet: A ROS-Enabled Multi-Sensing Platform for the ORCA Hub

**DOI:** 10.3390/s18103487

**Published:** 2018-10-16

**Authors:** Mohammed E. Sayed, Markus P. Nemitz, Simona Aracri, Alistair C. McConnell, Ross M. McKenzie, Adam A. Stokes

**Affiliations:** 1 School of Engineering, Institute for Integrated Micro and Nano Systems, The University of Edinburgh, Scottish Microelectronics Centre, Alexander Crum Brown Road, King’s Buildings, Edinburgh EH9 3FF, UK; m.mohammed@ed.ac.uk (M.E.S.); m.nemitz@ed.ac.uk (M.P.N.); simona.aracri@ed.ac.uk (S.A.); alistair.mcconnell@ed.ac.uk (A.C.M.); r.m.mckenzie@ed.ac.uk (R.M.M.); 2 Engineering and Physical Sciences Research Council (EPSRC) Centre for Doctoral Training (CDT) in Robotics and Autonomous Systems, School of Informatics, The University of Edinburgh, Edinburgh EH9 3LJ, UK

**Keywords:** communication fail-over, fault diagnosis, Limpet, on-board processing, ORCA Hub, real-time condition monitoring, remote sensing, robots, robot sensing systems, ROS Interface

## Abstract

The oil and gas industry faces increasing pressure to remove people from dangerous offshore environments. Robots present a cost-effective and safe method for inspection, repair, and maintenance of topside and marine offshore infrastructure. In this work, we introduce a new multi-sensing platform, the Limpet, which is designed to be low-cost and highly manufacturable, and thus can be deployed in huge collectives for monitoring offshore platforms. The Limpet can be considered an instrument, where in abstract terms, an instrument is a device that transforms a physical variable of interest (measurand) into a form that is suitable for recording (measurement). The Limpet is designed to be part of the ORCA (Offshore Robotics for Certification of Assets) Hub System, which consists of the offshore assets and all the robots (Underwater Autonomous Vehicles, drones, mobile legged robots etc.) interacting with them. The Limpet comprises the sensing aspect of the ORCA Hub System. We integrated the Limpet with Robot Operating System (ROS), which allows it to interact with other robots in the ORCA Hub System. In this work, we demonstrate how the Limpet can be used to achieve real-time condition monitoring for offshore structures, by combining remote sensing with signal-processing techniques. We show an example of this approach for monitoring offshore wind turbines, by designing an experimental setup to mimic a wind turbine using a stepper motor and custom-designed acrylic fan blades. We use the distance sensor, which is a Time-of-Flight sensor, to achieve the monitoring process. We use two different approaches for the condition monitoring process: offline and online classification. We tested the offline classification approach using two different communication techniques: serial and Wi-Fi. We performed the online classification approach using two different communication techniques: LoRa and optical. We train our classifier offline and transfer its parameters to the Limpet for online classification. We simulated and classified four different faults in the operation of wind turbines. We tailored a data processing procedure for the gathered data and trained the Limpet to distinguish among each of the functioning states. The results show successful classification using the online approach, where the processing and analysis of the data is done on-board by the microcontroller. By using online classification, we reduce the information density of our transmissions, which allows us to substitute short-range high-bandwidth communication systems with low-bandwidth long-range communication systems. This work shines light on how robots can perform on-board signal processing and analysis to gain multi-functional sensing capabilities, improve their communication requirements, and monitor the structural health of equipment.

## 1. Introduction

### 1.1. Challenges Facing Offshore Industries

The international offshore energy industry currently faces the challenges of: a fluctuating oil price, significant and expensive decommissioning commitments for old infrastructure, and small margins on the traded commodity price per kWh of offshore renewable energy [1]. Furthermore, the number of people available to work in offshore industries has reduced because graduates are shifting to less hazardous places onshore, and the current workforce is ageing. As the oil and gas requirements increase with the rapid growth of the world’s population, advanced technologies will become vital, and the cost of operation of these technologies will significantly rise due to the harsh and inaccessible nature of the environment. There have been several reported accidents and explosions of offshore rigs, with the most widely reported tragedy being the Deep Horizon oil spill in the Gulf of Mexico [2], which triggered the biggest debate from governments, academia, environmentalists and major companies of the oil and gas industry to look for safer ways to inspect, repair and maintain offshore platforms [3].

### 1.2. Application of Robotics in Offshore Industries

Operators are seeking more cost-effective and safe methods for inspection, repair, and maintenance of their topside and marine offshore infrastructure. Robots are seen as key enablers in this regard. Robots can be deployed in the air, on the rig or in the subsea, where they can be used to remotely monitor pipelines, corrosion, natural gas leaks, equipment conditions and real-time reservoir status. Hence, they can improve health, safety, and environment (HSE) and increase the production and cost efficiency.

### 1.3. Offshore Robotics for Certification of Assets (ORCA Hub)

**Figure 1:**
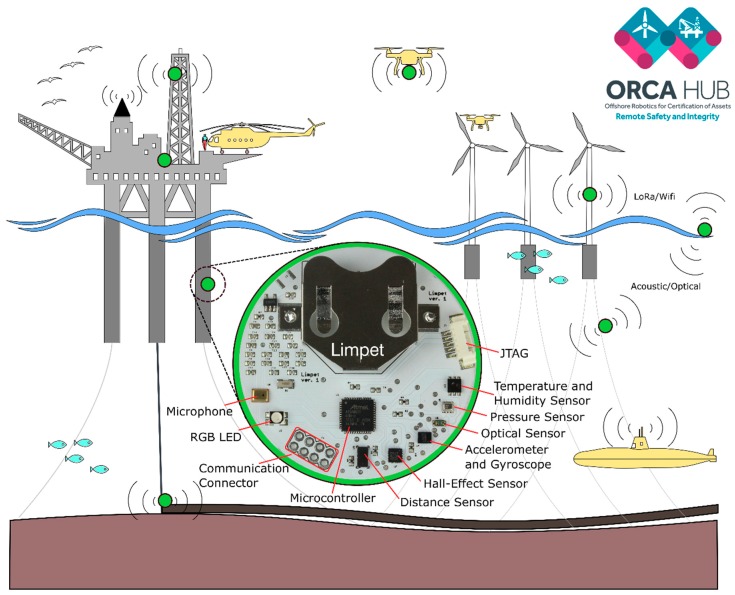
A conceptual overview of the ORCA Hub System. The Limpet, a part of the heterogeneous collection of robots that comprise the system, is shown in detail. The field robots and the Limpet communicate to each other, and the ORCA System controller, using Robot Operating System (ROS).

The UK Robotics and Artificial Intelligence Hub for Offshore Robotics for Certification of Assets (ORCA Hub) is a 3.5 year EPSRC funded, multi-site project with a vision to use teams of robots and autonomous intelligent systems (AIS) on remote energy platforms to enable cheaper, safer and more efficient working practices [1]. The ORCA Hub brings together top industrial companies (Total, Chevron, BP, and many others) with academia (University of Edinburgh, Heriot-Watt University, University of Oxford, Imperial College London, and University of Liverpool). The long-term vision for the ORCA Hub is an offshore platform that is completely autonomous and being operated and inspected from the shore, which will decrease the number of people working offshore. The ORCA Hub System constitutes the offshore platform, assets, sensors, and the heterogeneous robotic systems (topside, aerial, and marine robots) interacting with the platform as shown by Figure 1. In abstract terms, the proposed system is a sensor network with static nodes (Limpet agents) and mobile nodes (UAVs, drones, legged robots). We used Robot Operating System (ROS) as the software middleware for the proposed system, and, as such, the different nodes within the ORCA Hub System are referred to as ‘robots’ in this work following the concepts defined in ROS.

The ORCA Hub System provides remote solutions using robotics and Artificial Intelligence (AI) that are readily integratable with existing and future assets and sensors, and that can operate and interact safely in autonomous or semi-autonomous modes in complex and cluttered environments [1]. The ORCA Hub System aims at achieving tasks that are not possible with a single robot due to the complex nature of offshore platforms. It includes many components such as robotic autonomy, mobility, manipulation, sensor processing, autonomous mapping, navigation, multimodal interfaces, and human-machine collaboration. In this work, we focus on the sensing aspect of the ORCA Hub System. Sensing is a key element to the ORCA Hub System as it is important in asset monitoring, fault detection, mapping, environmental monitoring and helping other robots navigate around the platform.

### 1.4. Offshore Robotics (Current-State-of-the-Art and Challenges)

Robots on offshore platforms face many challenges. Some of these challenges include the absence of protection against saltwater spray and direct sunlight, harsh weather conditions involving rain, hail and high speed winds, extreme ambient temperature, high humidity levels, and dangerous atmospheric conditions resulting from gases, which can be explosive, toxic and corrosive [4]. For a robot to be suitable for operation on offshore platforms, there are some requirements regarding hardware, software, and communication. Some of the hardware requirements for offshore robots include making the electronics suitable for harsh environments and equipping the robot with highly reliable sensors to perceive its surroundings [4]. An example of the standards that an offshore robot must comply with is the ATEX directive 2014/34/EU (Appareils destinés à être utilisés en ATmosphères EXplosibles) in the European Union, DSEAR (The Dangerous Substances and Explosive Atmospheres Regulations of 2002) in Britain, and HAZLOC (Hazardous locations) in the United States of America. These standards cover any piece of equipment that is intended to be used in a potentially explosive atmosphere. Offshore robots also have requirements for software development including the ability to control the robot from remote locations, ease of programming new inspection and manipulation tasks without the use of experts, and the capability to alert remote operators when sensor values detected are abnormal [4]. Communication requirements mainly includes the ability to communicate and transmit data to a remote control station or to neighboring systems. As a result, of the harsh offshore conditions, remote sensing is becoming extremely valuable. Recent advances in the Internet of Things (IoT) and the cloud computing model provide support for remote sensing [5].

Currently, the majority of offshore robotics are used for subsea tasks, such as mapping of the seabed and inspecting underwater equipment and pipelines using AUVs and ROVs [6,7,8,9,10,11,12]. However, offshore robots for topside operations have started to gain the interest of researchers in recent years. Bengel et al. [13] were the first to demonstrate the application of robots for inspection and maintenance tasks in offshore platforms with their work on the MIMROex inspection robot. The Sensabot [14], which is developed by the National Robotics Engineering Center, is a tele-operated offshore inspection robot designed to withstand extreme weather conditions and harsh environment. Kyrkjebo et al. [15] developed the SINTEF Topside Robotic System, which can enable operators to control and monitor processes on the platform from onshore. Another robotic system developed by Galassi et al. [16] for offshore inspection and monitoring of offshore platforms is DORIS. DORIS moves through a rail and carries different sensors, where it can process the sensor data or store it for future analysis. The sensors can identify abnormalities such as intrudes in restricted areas, smoke, fire, liquid and gas leakages [16]. The robot has an embedded manipulator that allows it to take samples and read instruments. Walloid, which is a 4-arm quasistatic robot designed for offshore platform topside automation developed by Moghaddam et al. [17], is capable of climbing walls of different materials. In 2009, Bengel et al. [18] developed another mobile offshore inspection robot equipped with a robotic arm, a camera and several application sensors such as a microphone, gas sensor and fire sensor. The robotic prototype can autonomously execute pre-programmed inspection tasks in automatic mode.

### 1.5. Fault Detection and Condition Monitoring Systems

A potential application for robots on offshore platforms is fault detection and condition monitoring systems. Condition monitoring techniques help describe types of faults that can occur to a system, and the observable signs generated by these faults. Offshore implementation of fault detection and condition monitoring is challenging due to the complexity of remote sensing, data collection, data processing and data analysis. Condition monitoring systems play an important role in reducing maintenance and operational costs in offshore structures and, therefore, real-time condition monitoring and fault detection is essential [19,20].

In this work, we show a use case of condition monitoring for offshore wind turbines. However, the approach presented here is applicable to other offshore structures in oil and gas platforms such as rotating machinery, gears valves and cooling fans. Different techniques for condition monitoring of wind turbines have been proposed in the literature [21,22,23,24,25,26]. Some of these techniques for fault diagnosis of wind turbines are based on piezoelectric impact sensors [27], acoustic emission sensors [28], fiber Bragg grating sensor system [29,30], piezoceramic and impedance sensing [31,32]. However, real-time condition monitoring of wind turbines and fault detection has not been studied in detail, due to the harsh environments surrounding wind turbines, which makes experimental research challenging. Our work introduces a novel method to achieve real-time condition monitoring and fault detection for offshore wind turbines by combining signal-processing methods with remote sensing. The reliability of the monitoring system is enhanced by conducting all the data processing and analysis on the microcontroller, and transmitting data using high-range low-bandwidth communication systems only in the event of the presence of a fault.

### 1.6. The Limpet

In this work, we introduce our new multi-sensing platform, the Limpet, which is low-cost and designed for manufacturability. Our system is inspired by patella vulgate [33], commonly known as Limpet, which is an aquatic snail with a conical shell that lives on rocks on land or in the sea. It has a strong shell that protects it from the external environment and predators, and it can adhere to surfaces for long periods of time. The patella vulgata has an optic vesicle at the end of its tentacles that provides sensory function, and allows it to sense light and darkness levels [33]. We developed our bio-inspired system, the Limpet, for inspection and monitoring of offshore energy platforms. We embedded permanent magnets in the protective housing of the Limpet to enable it to attach to metallic surfaces. The protective housing acts as a shell that can protect the circuitry from the harsh offshore weather conditions. We can also integrate the Limpet with the ROS. The integration of the Limpet with ROS is essential, as it allows the Limpet to communicate with other robots that constitute the ORCA Hub system. The Limpet is designed to be one part (sensing) of the larger ORCA Hub System, where the system aims at realizing complex tasks that would not be possible with a single robot system.

We show how the Limpet is modelled as an instrument of sensing and measurement in Section 2.1. We discuss the design of the Limpet and how we integrate it with ROS in Section 2.2 and Section 2.3, respectively. We include a detailed description of the experimental setup we designed to conduct all our experiments in Section 3.1. We describe the offline and online classification of faults using the Limpet and the signal-processing components involved in these classifications in Section 3.2 and Section 3.3, respectively. We give an overview of the different communication strategies involved in this work and how we managed to achieve each strategy in Section 3.4. We give an overview of the signal processing and analysis tools used in this work in Section 3.5. We demonstrate and discuss the results of the experiments conducted in Section 4. We discuss how our approach can be applied to other sensors and other faults in Section 5.1 and Section 5.2, respectively. We compare on-board computation to offline computation in Section 5.3. We describe the different parameters that affect our measurements and explain how we try to reduce them in Section 5.4.

## 2. Design and Fabrication of the Limpet

### 2.1. Instrument Model

**Figure 2:**
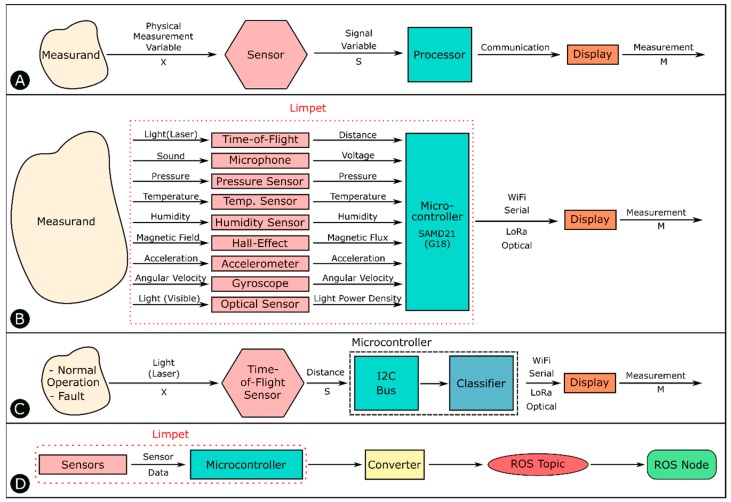
(**A**) Instrument model. (**B**) The Limpet system overview showing the nine sensing modalities and their corresponding signal variables. The Limpet can use several communication strategies to transmit the signal variables. (**C**) Instrument model for the Limpet for distance-based fault detection. We detect normal operation or fault measurands by using a time-of-flight sensor that converts the time of arrival of the reflected light into a distance measurement. (**D**) Architecture of the Limpet interface with ROS.

The instrument model [34] is a simple generalized model accepted by the sensors community to describe an instrument or a measuring device. In abstract terms, an instrument is a device that transforms a physical variable of interest (measurand) into a form that is suitable for recording (measurement) as shown by Figure 2A. An example of a basic instrument is a ruler. In this case, the measurand is the length of some object, and the measurement is the number of units (meters, inches, etc.) that represents the length.

The measurand (physical process) is represented by an observable physical measurement variable (X). There is a wide range of physical measurement variables. Common physical measurement variables include: force, temperature, humidity, acceleration, velocity, pressure, frequency, time, length, capacitance, resistance, etc. It is important to know that the physical measurement variable (X) does not necessarily have to be the measurand but can simply be related to the measurand. For example, the mass of an object is often measured by the process of weighing. In this case, the measurand is the mass but the physical measurement variable is the downward force the mass exerts in the Earth’s gravitational field. Another example is vibration detection. A robot can detect the measurand vibration by measuring force or by relating the measurand to another physical measurement variable such as acceleration. Both implementations will allow the detection of vibration; however, the accelerometer is much cheaper than the force sensor. There are also other situations where a single measurement variable contains information about multiple measurands. We refer to this capability as multi-functional sensing. One example is Infrared (IR) light. IR light has been used by robots for proximity measurements and communication with other robots [35]. Receiving signals from an IR transmitter requires sensors, such as photodiodes that transduce IR light into electric signals. Therefore, communication using IR light can be considered a sensing task. Another example is the use of the magnetic flux density from a single hall-effect sensor to detect whether the robot had a successful movement, collision or rotation measurands [36].

The key element of the instrument model is the sensor. The function of the sensor is to convert the physical measurement variable X into a signal variable S. Signal variables can be manipulated in a transmission system, such as an electrical or mechanical circuit, which means that they can be transmitted to an output device that is remote from the sensor. In our work, we use this property to be able to transmit the signal variable to a remote device using different communication or transmission systems. Typical signal variables include voltage displacement, current, force, frequency, light, pressure, etc. The output from the sensor is transmitted to a display or recording device, and the observed output is known as the measurement (M).

### 2.2. Design of the Limpet

#### 2.2.1. System Design

We equipped the Limpet with nine exteroceptive sensing modalities. The sensors incorporated in the Limpet are temperature, pressure, humidity, optical, distance, sound, magnetic field, accelerometer, and gyroscope. We conducted a literature survey to understand what parameters are relevant to offshore environments and monitoring of offshore structures and chose the sensors accordingly. We also got input from the industrial partners involved in the ORCA Hub project on the parameters that are interesting to monitor for the offshore platforms. These discussions influenced the choice of sensing modalities on the Limpet system. The main purpose of the Limpet is to monitor the offshore assets and the environmental conditions surrounding them. The Limpet can be modelled as an instrument, where each of its sensors converts a physical measurement variable into a corresponding signal variable as shown by Figure 2B. The signal variables are fed to the on-board microcontroller, which can then transmit the signal variable using one of several communication systems. In this work, we give one example of the multi-functional capabilities of the Limpet. Table 1 describes how the physical measurement from each sensor on the Limpet can be related to different measurands on offshore platforms. The table is based on the instrument model described in Figure 2A. It gives an example of a few potential measurands, and not all the measurands possible with each sensor, and it gives an overview of the multi-functionality that can be achieved with each sensor on the Limpet.

In this work, we demonstrate fault detection in equipment, specifically in wind turbines. We achieve the fault detection using a distance sensor, which is a Time-of-Flight (ToF) sensor. Not only does the distance sensor achieve condition monitoring and fault detection remotely, it gives an extra parameter, distance, which can be extremely useful in different applications (e.g., blade deflection monitoring), or when integrating it with other robots. The approach we used requires a sensor that is capable of remotely detecting the mechanical rotation of the wind turbine. For example, this approach can be performed using a magnetic sensor to measure magnetic reluctance. However, using a magnetic sensor would require the blades to be metallic. An accelerometer could also be used, by attaching it to the blade to monitor the speed and vibration, but the number of faults that can be detected are limited. A combination of a photodiode and phototransistor is another way to achieve fault detection in wind turbines, but it requires a complex setup and would only give indication of one parameter, which is absence of blade.

Figure 2C indicates the instrument model for the Limpet as considered in this work. The measurands normal operation and fault can be related to the physical measurement variable light. We use the distance sensor on the Limpet, which converts the physical measurement variable light to a signal variable distance, which is fed into the microcontroller’s I^2^C bus. We use the microcontroller to process the sensor data on-board and check them against a classifier. We use the distance measurements to classify if the machine is operating normally, or if there is a fault in the machine hindering its performance. The Limpet can send the data to a PC using one of several different communication systems. We can then use spectral analysis on the data to further reduce the information density and classify the type of fault detected.

The primary communication method of the Limpet is Wi-Fi. The Limpet uses Wi-Fi to transmit its data to a PC. We incorporated an ESP8266, which is a System-on-a-Chip (SOC) Wi-Fi module with integrated TCP/IP protocol stack capable of giving any microcontroller access to the Wi-Fi network, on the Limpet. We use Mosquitto [37], which implements a messaging protocol known as Message Queuing Telemetry Transport (MQTT), to send the data wirelessly from the Limpet to the PC. MQTT is a light-weight publish/subscribe messaging protocol used for remote communication. The received data can then be plotted in a real-time basis using MATLAB or saved on the PC and processed later.

We designed the Limpet to have robust communication. We can use multiple communication methods with the Limpet, including serial, Wi-Fi, LoRa and optical communication. LoRa is a digital wireless data communication technology that enables long-range transmission with low power consumption [38]. LoRa technology is provided by the LoRa Alliance, which is a non-profit association of more than 500 member companies that are developing and promoting LoRaWAN open standard for IoT. We achieve optical communication with a combination of the RGB LED and the optical sensor on the Limpet. Wi-Fi and serial communication do not allow agent-to-agent communication, unlike LoRa and optical communication. We incorporated a communication fail-over mechanism on the Limpet. If the primary communication method (Wi-Fi) fails for any reason, the functions of the Wi-Fi are assumed by a secondary communication module, which makes the system more fault-tolerant and robust in communication and data transmission.

The Limpet has an on-board programming port (JTAG). We programmed the Limpet using a SEGGER J-Link programmer together with a JTAG Adapter (Olimex ARM-JTAG-20-10). We programmed it in C/C++ using Atmel Studio 7. We used the standard Universal Asynchronous Receiver-Transmitter (UART) protocol for communication. The UART protocol uses a high idle line, which is pulled low at the start of a message.

Cost and functionality were the most important factors that we considered when designing the Limpets. Our rationale behind the system design was to keep the costs as low as possible without sacrificing functionality. The Limpet has a diameter of 50 mm, a height of 7 mm and weighs 17 g with, and 10 g without, the battery. We designed the Limpets to have a size and weight ideal for ease of fabrication, manufacturing, and assembly. The total cost of electronic components used in the design of the Limpet is about £22.

#### 2.2.2. Electrical Design

The Limpet consists of a single two-layer Printed Circuit Board (PCB) and a detachable Li-Ion coin cell battery. We designed a fully integrated PCB incorporating a low–power microcontroller (ATSAMD21G18A), RGB LED (LTST-N683EGBW), battery holder (BK-877) for a rechargeable Li-ion battery (LIR2477), charging IC (MCP73812T), charger connector, programming port [JTAG] (Molex 532610571), and a communication connector as shown by Figure 1. The PCB includes several exteroceptive sensors, which are: Temperature and Humidity Sensor (Si7006), IMU [Accelerometer and Gyroscope] Sensor (LSM6DS3), Optical Sensor (VEML6040), Sound Sensor (SPU0414HR5H-SB), 3-Axis Magnetic Sensor (MLX90393), Pressure Sensor (BMP280), Distance Sensor (VL53L0). Figure S1 shows the PCB schematic. The communication connector is connected to UART of the microcontroller. Therefore, we can use this connector to connect the Limpet to different communication systems. We designed the Limpet to use a single PCB for control, communication, and sensing.

The sensors on the Limpet, except for the microphone, are controlled by the microcontroller through the I^2^C bus. The microphone is an omnidirectional Micro-Electro-Mechanical System (MEMS) sensor, with an analogue output and a frequency range of 100 Hz to 10 KHz. In this work, we use the distance sensor for fault detection. The distance sensor on the Limpet is the smallest range sensor on the market today. It is a ToF laser-ranging module that can measure sub-mm distances for a range between 0 and 2.2 m. We also demonstrate the use of the optical sensor for local communication between two Limpets.

The Limpet could be used in a wide variety of areas, which leads to a wide range of temperatures that the components must be tolerant too. The datasheets of the sensors used show that they can be used in a wide range of temperatures. We designed the Limpet for the North Atlantic region which has a water temperature range of between 6 °C and 17 °C. This temperature range was within the allowed range of all the components used on the Limpet. The sensors used on the Limpet also have a wide humidity range. Some of the sensors used are splash resistant such as the IMU, hall-effect, and optical sensors. If we were to deploy the Limpet for field tests, it would require encapsulation to ensure resistance to extreme temperature and humidity conditions and saltwater spray. As noted in Section 1.4, the casing would have to comply with the regulations used in the specific area it would be deployed in.

We power the Limpet by a rechargeable 3.6 V 160 mAh lithium-ion coin cell battery. We can recharge the battery using a 6Vdc plug-in power supply. We included header pins on the PCB for connecting the power supply to recharge the battery. The Limpet has a battery life @160 mAh of 0.87 to 1600 h. We calculated the minimum battery life by assuming the Limpet has all the sensors, RGB LED, microcontroller and Wi-Fi communication continuously on. The Limpet will consume approximately 182.9 mA (ESP8266 consumes 135 mA, RGB LED consumes 20 mA, sensors consume 20.9 mA, microcontroller consumes 7 mA), which allows for a battery life of about 0.87 h or 52.2 min. We calculated the maximum battery life by assuming the Limpet is in sleep mode, where it consumes an average current of 0.1 mA. In this mode, the battery life of the Limpet can reach about 1600 h or 67 days. In the application presented in this work, the Limpet battery life is expected to last an average of 20 h if it is used for continuous monitoring. If we program the Limpet to sample once every hour, the battery life can go up to an average of 120 h.

#### 2.2.3. Mechanical Design

We designed the PCB of the Limpet using Eagle PCB Design Software and fabricated them on double-sided Cu-FR4-Cu 0.1 mm boards using an external company called Minnitron Ltd. (Kent, UK). We purchased all electronic components from RS Components and Digi-key Electronics. We soldered the components on the PCB using a reflow soldering process. In this process, we cut solder paste stencils from vinyl using a Laser Cutter (Epilog Laser Fusion 32).

We fabricated the protective housing from an optically clear, semi-rigid polyurethane resin. We developed the housing by casting the resin in a 3D printed mold. We did not cover the surfaces of the sensors by resin to keep them exposed to the external physical measurement variables. A picture of the encapsulated Limpet and the mold can be found in Figures S2 and S3.

We designed the Limpet explicitly for manufacturability; it consists of a single PCB and therefore mass manufacture is a simple case of placing a batch order with a PCB foundry. The PCB consists of surface mount components, except for the communication connector, and can be autonomously populated with pick-and-place machines at the point of manufacture. Systems that are made of a single PCB with mostly surface components allow for scaling up to huge collectives of agents easily without sacrificing functionality [39]. Assembly of one Limpet takes seconds as it is a matter of only connecting the coin cell battery. Once the battery is connected, there is no need to remove the battery from the Limpet again as it can be charged on-board. As a result, of the Limpet being highly manufacturable and easy to assemble, it is easy to mass-produce Limpet agents and deploy them in huge collectives.

### 2.3. ROS Interface

We can interface the Limpet with ROS, and the architecture of this interface is shown by Figure 2D. The sensor data are fed into the microcontroller, and the microcontroller then sends out the data to a converter. The converter is a custom-written python script on the remote PC, which receives the data from the Limpet through one of the communication strategies (Wi-Fi/LoRa/serial) and converts this data into a ROS protocol. We can publish this data to a ROS topic, and any ROS node can subscribe to that topic to read the sensor data. The different sensors on the Limpet have different physical measurement variables. When these measurement variables are fed into the microcontroller, it adds a label to the data to differentiate data from the different sensors (e.g., distance, temperature, pressure, etc.). The data are published to the relative ROS topic based on this label as shown by the overview in Figure S4. ROS nodes can subscribe to relevant topics to gain access to the sensor data. Interfacing the Limpet with ROS enables it to interact with other robots in the ORCA System that are running ROS, where the interaction between them can result in a more complex and useful behavior. Figure S5 depicts the results from integrating the Limpet with ROS. It shows a screenshot of the data published to the distance ROS Topic, and a graph of the distance measurement from the ROS Topic during normal operation of the fan.

## 3. Experimental Design

The work presented here focuses on the sensing capabilities of the Limpet. In this work, we use the ToF sensor to demonstrate how distance measurements can be used to detect faults in the operating conditions of machines or equipment. We show how a measurement variable can be indirectly related to the measurand and demonstrate an example of how the Limpet could be used for condition monitoring. The approach presented in this work can used for monitoring different offshore structures. This section discusses the design of the experiments that we used to demonstrate the capabilities of a single Limpet in fault detection and condition monitoring.

### 3.1. Design of the Distance-Based Fault Detection Experimental Setup

**Figure 3:**
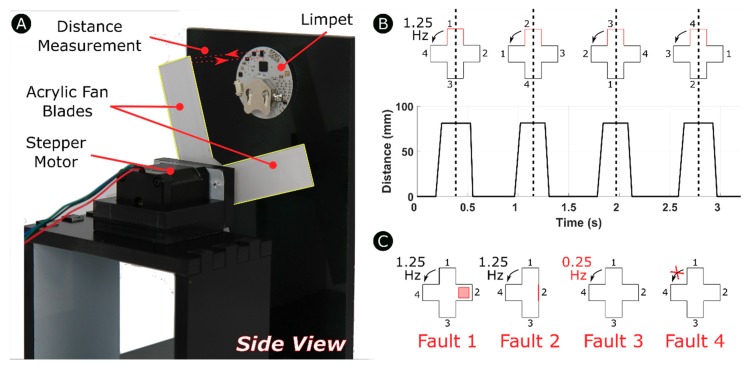
(**A**) Experimental setup for distance-based fault detection. We fixed the Limpet in place and used the fan to mimic a small wind turbine. (**B**) Schematic of the fan and distance profile for normal operation mode. Each peak corresponds to one of the four fan blades detected. (**C**) Schematic of the faults introduced to the system.

The experimental setup consists of a platform containing the Limpet and a custom-designed fan with four blades rotating in front of the Limpet as shown by Figure 3A. We designed the fan blades by cutting a pattern out of an acrylic sheet using a laser cutter. We attached the fan blades to a stepper motor to achieve rotation. We used the fan to mimic a small wind turbine. We placed the fan 85 mm from the Limpet. We use the ToF sensor on the Limpet to measure distance to the fan blades as they are rotate. We controlled the speed of rotation of the fan by controlling the speed of rotation of the stepper motor. When a blade passes in front of the Limpet, the blade is intercepted by the ToF sensor, and a distance measurement is recorded. We programed the Limpet to interpret the absence of a blade as a zero measurement. Figure 3B shows a schematic of how the distance measurement was determined for the fan for normal operating conditions. It shows the distance profile, which depicts the distance measurement for each blade passage in front of the Limpet. The frequency of detection of the fan blades is 1.25 Hz under normal conditions. The fan has four blades, and each blade corresponds to a peak on the graph. In our case, the zero value represents the absence of a blade in front of the Limpet. We recorded the distance profile over a period of 3.2 s, which corresponds to the frequency of rotation of the fan. The difference in width between the peaks is due to the error in detection of the edges of the blade, which is discussed further in Section 5.4.

To evaluate the capability of distance-based fault detection using the Limpet, we divided the work into two parts: offline classification and online classification of faults. We tested the offline classification approach using two different communication techniques: serial and Wi-Fi. We performed the online classification approach using two different communication techniques: LoRa and optical. We conducted five experiments for each of the four communication systems. We conducted each of the five experiments separately for each communication system. We conducted all the experiments under normal laboratory conditions. We designed one of the five experiments to be normal operation of the fan. In the other four experiments, we introduce a fault to the system and check if the Limpet is capable of detecting this fault. Thus, we tested the detection of the fault using both approaches (offline and online classification). The faults introduced to the system are listed below and a schematic of these faults are shown by Figure 3C.

- **Fault 1:** We attach an object of 15 mm thickness to one of the four blades.
- **Fault 2:** We remove one of the four blades of the fan.
- **Fault 3:** We reduce the frequency of detection of a fan blade from 1.25 Hz to 0.25 Hz.
- **Fault 4:** We immobilize the rotation of the fan for a certain period during its normal operation.

### 3.2. Design of the Offline Classification Experiments

**Figure 4:**
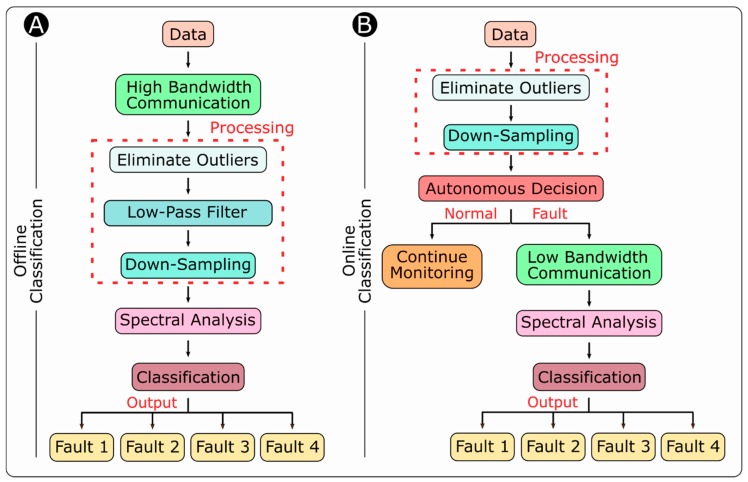
Overview of the signal-processing components for (**A**) Offline Classification and (**B**) Online Classification.

Figure 4A gives an overview of the components included in the offline classification process of the system. First, we conduct the distance measurement experiment with the Limpet and record the data (distance measurement, timestamp). We transmit the data from the Limpet to the PC in a real-time manner using a high-bandwidth communication system such as serial or Wi-Fi communication. Since there is no memory limitation, as the data are not stored on the microcontroller in the offline classification approach, we recorded the sensor data over a period of 300 s. We then process the data on the PC using MATLAB. We apply a Median Absolute Deviation (MAD) algorithm to remove any outliers from the dataset. We then apply a low-pass filter (simple moving average) to the data to remove the white noise. We also down-sample the data after application of the low-pass filter. In the presence of a fault, given this processed dataset, we apply spectral analysis to classify the type of fault. This offline classification process is used as a learning basis for online classification on the Limpet.

To test for other faults, we conducted two additional experiments using Wi-Fi and serial communication (offline classification); these were: sampling at a rate lower than the Nyquist Frequency, and immobilizing the fan twice during the 300 s measurement period.

When we conducted the experiments for Wi-Fi and serial communication on normal operation of the fan, we calculated the average and standard deviation of the distance measurements recorded over a period of ten min. We used these values as learning values for the microcontroller when performing the online classification process.

### 3.3. Design of the Online Classification Experiments

Figure 4B gives an overview of the online classification process. In the online classification approach, the Limpet does all the processing and analysis on-board, and then takes an autonomous decision based on the classification result. The Limpet starts sampling the distance to the fan using the ToF sensor. The Limpet stores several distance measurements into its memory (120 measurements). The Limpet processes the data, by first removing any outliers from the sensor data. The data are then down-sampled according to the down-sampling frequency to accord with the limitations of the microcontroller on the Limpet. The Limpet classifies the dataset using a sliding window algorithm. The sliding window algorithm is used to process data streams, where the input is presented as a sequence of items, and the function of interest is computed over a fixed-size window in the stream [40,41]. The Limpet detects the first rising edge and the fourth falling edge and calculates the period between them. This period is checked against the frequency of normal operation stored in the microcontroller’s memory. The distance measurements are also compared to the average value and standard deviation of normal operation, which were stored in the microcontroller after training the classifier offline.

According to the offline training of the classifier, the distance measurement for each blade must fall within a range of average ± standard deviation. The parameters of the class representations (average, standard deviation, time period) were trained offline and do not change during operation. These parameters are stored on the Read-Only Memory (ROM) of the microcontroller. The amount of ROM used depends mainly on the number of points (120) in the dataset and their size (distance is 8-bit, time is 16-bit, distance index is 8-bit). After the Limpet checks the dataset for the presence of any faults, it makes an autonomous decision based on the result of the classification. Autonomy is the ability of a system to make its own decision and to act on its own without external intervention [42]. Within autonomy, there are many variations concerning where and how decisions are made, and actions are invoked. An automatic system carries out several fixed and prescribed activities, where there may be options, but they are fixed in advance and follow a rigid cycle [42]. An adaptive system uses feedback from the environment to improve its performance [42]. A completely autonomous system makes decisions based on the system’s view of the situation at the time of decision and its current view of the environment [42]. Decisions made by completely autonomous systems mimic human-level decisions.

Since the Limpet carries out several prescribed activities or checks during online classification, it can be considered an automatic system. If the dataset is within the specified range and frequency, the Limpet continues monitoring the system, but if the dataset fails any of the checks, the Limpet classifies the measurements as faulty operation. The Limpet then transmits the processed data using low-bandwidth communication systems such as LoRa and optical communication to the PC, where spectral analysis is performed to classify the fault detected. For optical communication, we placed another Limpet in front of the Limpet that is part of the distance-based fault detection experimental setup. We aligned the second Limpet such that it does not block the distance sensor of the first Limpet, but at the same time with its optical sensor aligned with the RGB LED of the first Limpet.

### 3.4. Overview of the Communication

In this work, we use four different communication methods (Wi-Fi, serial, LoRa, optical) to transmit the sensor data from the Limpet to the PC. Figure S6 gives an overview of the communication strategies involved in this work and how they are achieved. For serial and Wi-Fi communication, the communication bandwidth is high, and we can send the sensor data from the Limpet continuously in real time to the PC. We can then analyze the data on the PC using analysis tools such as MATLAB. In this regard, the Limpet is only making a measurement and transmitting it instantly to the PC without doing any processing on the data, which means serial and Wi-Fi communication are not computationally demanding on the Limpet. We transmit the data from the Limpet over Wi-Fi using the ESP8266, and serially using SparkFun’s FTDI Basic Breakout-3.3 V, which is a serial to USB convertor.

LoRaWAN is a new wide area network (WAN) technology that allows for long-range communication, but with a limited bandwidth. Low power WAN combines robust modulation and low data rate to achieve long-range communication. LoRaWAN is one of the most successful tools in low power WAN technologies. It is a network stack rooted in the LoRa physical layer. LoRaWAN features a low data rate (a maximum of 27 kb/s with spreading factor 7 and 500 kHz channel) and long-range communication (2–5 km in urban areas and 15 km in suburban areas). There is a tradeoff between the spreading factor (SF) and communication range in LoRa. The higher the SF, which means the slower transmission, the longer the communication. Depending on the spreading factor used (7 to 12), LoRaWAN data rate ranges from 0.3 kb/s to 27 kb/s [43]. LoRa has a much lower communication bandwidth than serial and Wi-Fi communication. Thus, when we use LoRa technology with the Limpet for communication, the limited bandwidth limits the ability to send the sensor data continuously in real time to the PC. Therefore, if the dataset is large, processing and analysis of the data need to be done before transmission to allow for only small amounts of data to be transmitted. This on-board processing and analysis approach requires higher computational power than serial and Wi-Fi communication. We used the LoPy, which is a MicroPython enabled Wi-Fi, Bluetooth and LoRa development board, to gain access to the LoRa network. The LoPy transmits the data from the Limpet to The Things Network, which is a network that uses LoRaWAN to allow for devices to talk to the Internet without 3G or Wi-Fi.

Optical communication has the lowest bandwidth among the four communication strategies. The processing and analysis also need to be done before the transmission of data. In our case, we transmitted data using the on-board RGB LED. We transmit the data by pulse-width modulating (PWM) the LED to different levels, where each level corresponds to a number from 0 to 9. We pulse-width modulate the RGB LED from 0 to 255 in steps of 25, which allows for ten different intensity levels corresponding to each of the numbers (0 to 9). We used the different LED colors to correspond to different measurements such as time, distance, and index of measurement. We can use the other LED colors for other sensor measurements such as pressure, temperature, etc. Our optical communication protocol transmits a single digit at a time. Therefore, we programmed the Limpet to divide all the measurements into single digits and transmit the digits one after the other. The intensity of the transmission (PWM level) and the LED color (measurement type) allows us to reconstruct the transmitted data from the RGB LED.

We used the optical sensor on the Limpet as a receiver. We used it to read the light intensity (indication of the transmitted number) and color power density (indication of measurement type), to interpret the data transmitted using the RGB LED. Before we conducted any experiments using optical communication, we ran a calibration experiment for the light sensor using two Limpets. We PWM the RGB LED from 0 to 255 in steps of 25 on one Limpet. We pulse-width modulated the red LED first, followed by the green LED and the blue LED. We used the optical sensor on the other Limpet to read the light intensity and color power density of the LED for each PWM level, and the results are shown by Figure S7. Plots of the power density versus time for different numbers transmitted using the red, green, and blue LED are shown by Figures S8, S9 and S10, respectively. We used the results of this calibration experiment as a reference to interpret the data transmitted by the Limpet during the experiments with optical communication. We used the red LED to send distance measurements, the green LED to send timestamps, and the blue LED to send the index of the data point. Figure S11 shows a plot of the power density from each LED for each of the numbers transmitted. The data transfer speed achieved in our optical communication lies in the range of 6 bps to 53 bps.

There is an extra step involved in the optical communication, which is the calibration and use of the optical sensor to infer the transmitted data. This extra step implies a higher computational power demand for optical communication. Optical communication cannot be used for long-range communication, where the optical communication range in this work is in the region of a few 10 s of cm. The optical communication range is a function of both the intensity of the RGB LED and the sensitivity of the optical sensor. Optical communication can be useful for communication with underwater vehicles, as it was discovered that blue light can propagate in seawater better than other frequencies of light [44]. In field conditions, the amount of ambient light intensity has an impact on the optical communication system.

### 3.5. Overview of the Classification Techniques

#### 3.5.1. Signal-Processing Tools

To eliminate outliers from the input signal, we applied a MAD Algorithm. MAD algorithm is used in statistics as a measure of the variability of a sample of quantitative data. MAD is more resilient to outliers in a dataset than the standard deviation. Since MAD is more robust to scale estimates than the standard deviation, it is better applied to data that is not normally distributed, which is the case in this work. Consider an input signal $x=\left[x_{1},\ldots ,\;x_{N}\right]$ with length N. MAD is defined as the median of the absolute deviations from the dataset’s median, and is given by

(1) $$\;\mathrm{MAD}=median\;\left(|x_{i}-\;median\left(x\right)|\right),\;i=1,\ldots ,N\;$$

In this work, we considered data points greater than 3 median absolute deviations from the dataset’s median as outliers, and we replaced them with the mean of the dataset.

After the successful elimination of outliers, we applied a low-pass filter to the data to remove white noise from the input signal. We applied a simple moving average filter to smooth out the short-term fluctuations in the data. In this stage, we calculate the mean from an equal number of data points on either side of a central value. By doing so, the variations in the mean are associated with the variations in the data instead of being shifted in time.

For an input signal *x* and a positive integer *k*, the moving average *y* is computed by sliding a window of length *k* along *x*. Each element of *y* is the local mean of the corresponding values in the input signal *x* within the window, and signal *y* is the same size as *x*. For a signal *x* made up of *k* scalar observations, the mean µ and the moving average *y* are given by

(2) $$\;µ=\frac{1}{k}\;\overset{k}{\underset{i=1}{\sum }}x_{i}\;$$

(3) $$\;y=\left[{µ}_{i},\ldots ,\;{µ}_{N}\right]\;$$

We have chosen a moving average sliding window of size (*k* = 8) as the distance sensor records 8 data points for each blade passage in front of the sensor due to sampling frequency we set for the sensor (25 Hz). The time series after application of the low-pass filter varies within a narrower range as compared to the signal after elimination of outliers.

After elimination of outliers and noise, we reduce the input signal $x=\left[x_{1},\ldots ,\;x_{N}\right]$ with length *N* in size to accord with the computational limitations of the microcontroller. We reduce the length of the signal (*N*) by dividing the signal into L parts, each of which contains $M=\frac{N}{L}$ measurements. The value of each element $x_{M}^{\prime }$ of the down-sampled signal $x^{\prime }$ is given by

(4) $$\;x_{m}^{\prime }=\frac{1}{M}\;\overset{Ml}{\underset{i=M\left(l-1\right)}{\sum }}x_{i},\;l=\;1,\ldots ,L\;$$

In this work, we down-sample the full distance measurement for one complete fan rotation to 8 measurements (*M* = 8) corresponding to the four peaks in the distance profile. We conduct the same process on the timestamp and index information sent with the original data. Down-sampling has an impact on the classifier’s detection rate. The higher the down-sampling frequency on the input signal, the worse the detection rate becomes. Down-sampling potentially removes important features in the signal that can help the classifier discriminate between signals constituting normal operation or a fault. However, the higher the down-sampling frequency, the lower the memory required to store values of the cumulative distance matrix. Memory reductions are useful since it lowers the requirements for our low-cost microcontroller. Therefore, there is a tradeoff between memory reduction and classifier performance. In this work, we were able to achieve good classification performance with suitable memory usage as shown in the Results Section. We determined the down-sampling frequency after conducting multiple experiments for normal operation of the fan and studying the signal for characterizing features in the distance profile. We conducted each experiment for a period of 300 s, during which we recorded 7322 data points. Among the recorded data, 802 data points corresponded to the detection of rising and falling edges, i.e., 401 peaks. Therefore, the down-sampling frequency is calculated to be ~9 (7322/802).

#### 3.5.2. Analysis Tools

After processing the input signal, we use spectral analysis to classify the fault detected. Spectral analysis has been used extensively in literature for fault detection [45,46]. We first apply a fast Fourier Transform (FFT) algorithm to the processed signal, which divides the time signal into its frequency components. These components are single sinusoidal oscillations at different frequencies, where each component has its own phase and amplitude. The FFT algorithm is the Discrete Fourier Transform (DFT) of a sequence. FFT computes the same results as DFT but can do it more quickly. In terms of time complexity, which is described as the amount of time needed to run an algorithm based on its computational complexity and expressed using big O notation, DFT takes **O(N^2^)** arithmetical operations, while FFT takes **O(N log N)** arithmetical operations for an input signal with *N* points. The *N*-point DFT equation for a finite-duration sequence *x*(*n*) is given by

(5) $$\;X\left(f\right)=\;\overset{N}{\underset{n=1}{\sum }}x\left(n\right)e^{-j\frac{2\pi }{N}\;\left(f-1\right)\left(n-1\right)},\;1\leq f\leq N\;$$

Following the application of the FFT algorithm, we compute the power spectral density function (PSD) of the signal. The PSD shows the variation of power as a function of frequency, which could be used to detect abnormal system behavior. Thus, observing the PSD of the recorded sensor data will enable classifying the fault using a single parameter. The power spectral density for a signal *x*(*n*) with *N* points is given by

(6) $$\;P\left(f\right)=\;\frac{1}{N}\;|X{\left(f\right)|}^{2}\;$$

For a periodic rectangular pulse function, the FFT divides the pulse signal into several harmonics (sine waves) to approximate the pulse signal. Adding more sine waves of increasingly higher frequencies improves the approximation of the rapid changes (discontinuity) in the pulse signal. In the frequency domain, a periodic rectangular pulse function results in a sinc() function centered around the dominant frequency. As the duty cycle or pulse-width of the signal is varied, the sinc() function changes. As the duty cycle is decreased, the width of the of the sinc() function broadens (i.e., it becomes less localized in frequency). Thus, if a function changes rapidly in time, the signal must contain high frequency component to allow for such a rapid change.

## 4. Results

### Distance-Based Fault Detection

**Figure 5:**
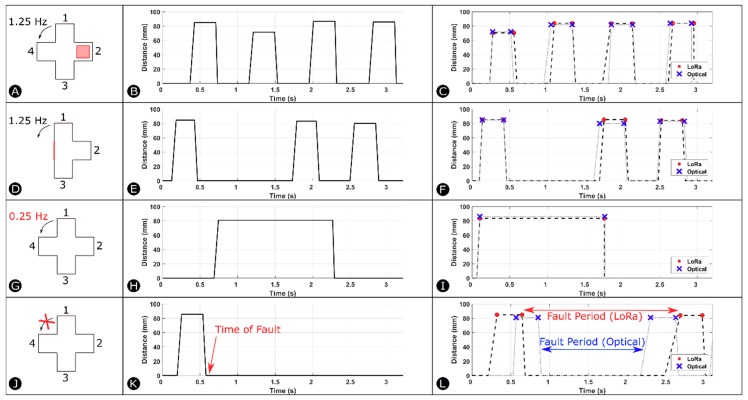
Schematic of (**A**) Fault 1, (**B**) Fault 2, (**C**) Fault 3, (**D**) Fault 4. Distance Profile of Wi-Fi communication experiment for (**E**) Fault 1, (**F**) Fault 2, (**G**) Fault 3, (**H**) Fault 4. Distance Profile of LoRa and optical communication experiments for (**I**) Fault 1, (**J**) Fault 2, (**K**) Fault 3, (**L**) Fault 4.

Figure 5 depicts the results of the experiments for Wi-Fi (offline classification), LoRa and optical communication (online classification). The results from the experiments we conducted using serial communication (offline classification) were similar to the results from the Wi-Fi experiments. Figure 5 shows the distance profile resulting from each of the four faults introduced to the system. We constructed the distance profile for LoRa and optical communication (dotted lines) from the down-sampled results of the experiments (markers). Every two consecutive points (markers) on the graph represent a single peak or a fan blade. The distance profiles are shown within a period (T) of 3.2 s (corresponding to the frequency of rotation of the fan during normal operation). When a fault is introduced to the fan and stepper motor system, its distance profile looks distinctly different as compared to the distance profile of normal operation. Each fault also possesses a distinctive power spectral density plot as compared to other faults.

Figure 5A shows a schematic of Fault 1. Figure 5E shows the distance profile acquired using Wi-Fi communication, and Figure 5I shows the distance profile for LoRa and optical communication, after we introduce Fault 1 to the system. As seen in both Figures, one of the peaks in the distance profile has an amplitude lower than the other peaks as a result of the object attached (extra thickness) to it. Figure 5B shows a schematic of Fault 2. The results for Wi-Fi communication, and LoRa and optical communication are shown by Figure 5F,J, respectively. The results of the three communication systems show that one of the peaks in the distance profile is missing as compared to normal operation. Figure 5C shows a schematic of Fault 3, where we reduced the frequency of detection of the fan blade to 0.25 Hz. The distance profile for Wi-Fi, LoRa and optical communication shows only a single peak within 3.2s as shown by Figure 5G,K, as the frequency of blade detection is lower than normal operation. Figure 5D shows a schematic of Fault 4, where we immobilized the fan during its normal operation. The distance profile for Wi-Fi communication shows the absence of any peaks after the introduction of the fault to the system as shown by Figure 5H. Figure 5L shows the down-sampled results and the distance profile for LoRa and optical communication. The graph shows that two of the peaks are missing corresponding to where the fault is present. The fault period is slightly different for LoRa and optical communication experiments. The fault duration is also different for LoRa and optical communication as compared to Wi-Fi communication. For Wi-Fi communication, we recorded the distance measurements for a long period, and, thus the fault was introduced over a larger time window.

The raw sensor data we recorded from the Limpet during each of the five experiments using Wi-Fi communication are shown by Figure S12. The data after elimination of outliers, application of a low-pass filter, and down-sampled results can be found in Figures S13, S14 and S15, respectively. For serial communication, the raw sensor data, data after elimination of outliers, results of the low-pass filter and down-sampled results can be found in Figures S16, S17, S18 and S19, respectively. Figure S20 shows the distance profile from the serial communication experiments within a period (T) of 3.2 s.

**Figure 6:**
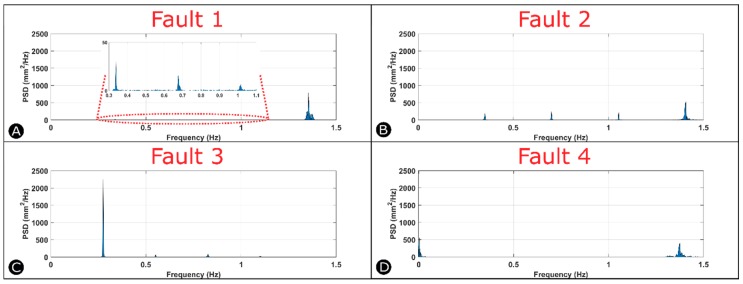
Spectral analysis of the data after introducing (**A**) Fault 1, (**B**) Fault 2, (**C**) Fault 3 and (**D**) Fault 4 to the system.

Figure 6 shows the power spectral density plots for each of the four faults. We obtained these results from the experiments using Wi-Fi communication. During normal operation, the spectral analysis gives a single output signal (peak) at a frequency of 1.3 Hz as shown by Figure S21, which corresponds to the dominating harmonic frequency that best describes the signal in the time domain. In the time domain, the distance profile is a periodic rectangular pulse signal, therefore, in the frequency domain the harmonics exist around the same frequency. This collection of similar harmonics results in a peak in the frequency domain that is distributed around the dominating frequency. Figure 6A shows the power spectral density graph resulting from the introduction of Fault 1 to the system. There is a large peak at a frequency of 1.3 Hz, and three smaller peaks at frequencies of 0.35 Hz, 0.7 Hz and 1 Hz. The peak at 1.3 Hz represents the dominant harmonic in the time domain signal, while the other peaks are present because of the harmonics introduced to the system due to the presence of Fault 1. The PSD divides the signal into its single sinusoidal components at the different frequencies. In this case, the time domain signal is more complex, since the pulse signal is not periodic anymore. Therefore, the harmonics in this signal are more complex and introduce lower frequency components to describe the signal in the frequency domain. Figure 6B shows the power spectral density graph resulting from the introduction of Fault 2 to the system. There is a large peak at a frequency of 1.3 Hz, and three smaller peaks at frequencies of 0.35 Hz, 0.7 Hz and 1.1 Hz. The peak at 1.3 Hz represents the dominating harmonic in the time domain signal, while the other peaks are present because of the harmonics introduced to the system due to the presence of Fault 2. The absence of the peak in the time domain signal introduces new harmonics in the spectrum represented by the lower frequency components. This power spectrum is distinct from the power spectrum of Fault 1, as the power in the lower frequency harmonics is higher due to the larger difference in the amplitude between the peaks in the distance profile. The spectral analysis of Fault 3 shown by Figure 6C depicts a single large peak at a frequency of 0.25 Hz, which corresponds to the lower frequency of detection of the fan blades. The signal is a periodic rectangular pulse signal, and thus, results in a spectral plot with a peak around the dominant frequency. Since the duty cycle is increased as compared to normal operation, the peak is narrower and larger in the frequency domain. The spectral analysis of Fault 4 shown by Figure 6D depicts a large peak at a frequency of 1.3 Hz representing the normal fan operation and another peak at a lower frequency representing the break of rotation of the fan. The amplitude of the peaks depends on the length of the jam introduced to the system. Figure S22 shows the power spectral density plots from the Wi-Fi experiments plotted against time.

Figures S23 and S24 show the power spectral density plots from the serial communication experiments plotted against frequency and time, respectively. The results of the spectral analysis on the serial communication experiments are similar to the spectral analysis of the Wi-Fi experiments. Figure S25 shows the optical sensor data, which is obtained from the transmission of measurements using the RGB LED during the optical communication experiments, and which shows the down-sampled results from the experiments and the spectral analysis performed on the data for both LoRa communication and optical communication after we introduce the four faults to the system. The frequency of the peaks in the power spectral density graph of LoRa and optical communication experiments are similar to those of serial and Wi-Fi experiments. The only difference is the amount of power or energy in each peak. The power in the sinusoidal oscillations is much higher in serial and Wi-Fi experiments as the results were recorded over a larger period. The results from the two additional experiments (jamming the fan twice and a lower rate of sampling) we conducted using Wi-Fi communication and serial communication are shown by Figures S26–S29.

## 5. Discussion

In this paper, we combined ideas from the fields of offshore robotics, remote sensing and signal processing and designed a system to demonstrate that low-cost hardware can be a useful tool to monitor offshore energy platforms. The signal-processing techniques we used in this work were explicitly adapted to operate on our low-cost hardware. We introduced a down-sampling method to reduce the time taken to run the classifier and to decrease the size required in memory on the microcontroller. We performed a sliding window algorithm on-board as a rapid test of the system’s functionality. The same approach can be applied to other faults and other sensors, which we discuss in Section 5.1 and Section 5.2, respectively. We discuss, in Section 5.3, the advantages and disadvantages of on-board computation as compared to offline computation. We discuss possible interferences to our measurements, and how we reduce them, in Section 5.4.

### 5.1. Applicability to Other Faults

The classification approach we adopted is a generic solution to condition monitoring and not specific to the faults we introduced to the system in this work. Our method depends on the change in normal operation conditions of equipment, where the change is associated with the presence of a fault in the system. We trained our classifier offline with conditions related to normal operation of the fan. The offline classification determines multiple parameters for normal operation including average distance to the fan blades, standard deviation of distance measurements, and frequency of rotation of the fan. These parameters are then transferred to the Limpet for online classification and do not change during operation. During the online classification, the Limpet checks the parameters of the class representations (average, standard deviation, period) to the parameters of normal operation stored on the microcontroller’s memory. If any of the parameters fails the check, the Limpet classifies the measurement as a faulty operation. Thus, any fault that changes the normal operation conditions of the fan, which in turn results in a change in these parameters, will be detected and flagged as faulty operation by the Limpet. We believe that our method is applicable to several different fault types. We test our approach by introducing four specific faults to the system to determine if the Limpet can detect these faults. Examples of other faults that could be detected for wind turbines or rotating machinery are bending of blades, change of distance between blades, or undesirable vibration or oscillation of blades.

### 5.2. Applicability to Other Sensors

In this work, we have shown how we can use a single sensor to monitor the state of equipment by measuring several measurands, as well as how we can process the data and analyze it on-board to improve communication requirements. Our method is dependent on distinct measurement profiles associated with each measurand. The method also depends on time-varying measurements. We think that this approach is applicable to other sensors and systems, if the measurement profile is (i) a time-varying measurement and (ii) distinctly different from other profiles. Thus, this method could be used by other sensors to identify different types of faults with equipment or to monitor different parameters on offshore platforms. It could also be easily adapted to other low-cost systems.

### 5.3. On-Board Data Processing vs. Offline Processing

By processing and analyzing data on-board using the microcontroller, we can improve the communication and power requirements of the system while still being able to perform same analysis. Data transmission does not need to occur continuously, but only in the case of a particular event. This design allows us to use communication systems with high transmission distance, but low bandwidth (such as LoRaWAN), and increase battery life of systems as the data transmission link is the most power-consuming part of a system. Offline processing involves continuously transmitting data and analyzing it to detect the presence of any faults. This approach is time-consuming, reduces battery life and requires high communication bandwidth, which lowers transmission distance. The major drawback of our approach is that low-cost hardware is limited in its computational power. As such, we are restricted to the types of algorithms that could be used on the system.

### 5.4. Mitigation of Interferences

By processing and analyzing data on-board using the microcontroller, we can improve the communication and power requirements of the system while still being able to perform same analysis. Data transmission does not need to occur continuously, but only in the case of a particular event. This design allows us to use communication systems with high transmission distance, but low bandwidth (such as LoRaWAN), and increase battery life of systems as the data transmission link is the most power-consuming part of a system. Offline processing involves continuously transmitting data and analyzing it to detect the presence of any faults. This approach is time-consuming, reduces battery life and requires high communication bandwidth, which lowers transmission distance. The major drawback of our approach is that low-cost hardware is limited in its computational power. As such, we are restricted to the types of algorithms that could be used on the system. Several parameters impact the sensor measurements and interferes with the classification process. Five of these parameters are: (1) Displacement of the Limpet during the experiment, which will affect the distance measurement to the equipment and will impact the classification process; (2) Change in the distance between the Limpet and the fan for the different set of experiments; (3) The target surface used for the fan blades. Different target surfaces will reflect light (laser) differently and might thus affect the distance measurements; (4) Presence of objects behind the experimental setup or fan. The distance sensor can detect distances up to 2 m, and the presence of any objects behind the fan would affect the distance measurement level (zero) for absence of fan blades; and (5) The Field of View (FoV) of the distance sensor, which is the angle at which the receiver on the sensor is sensitive to electromagnetic radiation. The distance sensor has a FoV of 25°, which causes it to detect the edge of the fan blade before the blade center is opposite the sensor. This error results in a larger number of distance measurements versus time as each blade passes in front of the limpet, and therefore, the blade appears wider than its actual width when observed by the receiver of the sensor.

In our work, we reduce the effect of these parameters on our measurements in five ways: (1) We fixed the Limpet in place on a small wall in front of the fan to reduce any displacement by the Limpet; (2) We fixed the stepper motor in place in the experimental setup to minimize the errors in measurement by having the fan always at the same distance from the Limpet for all the experiments; (3) We used a white acrylic surface for the fan blades, as white targets cause maximum reflectance; (4) We place an acrylic sheet at a distance of 2.5 m behind the fan and setup, which is out of the range of measurement of the distance sensor, and thus ensures the same measurement level for absence of blades; and (5) We compute the moving average of our sensor measurements with a sliding window of size (8) to reduce the effect that the FoV introduces on our measurements.

## 6. Conclusions

In this work, we introduce a new multi-sensing platform, the Limpet, which is part of the ORCA Hub System. We embedded the Limpet circuitry in a protective housing to protect it from the harsh offshore weather conditions. We have integrated the Limpet with ROS, which allows it to be used with other robots within the ORCA Hub System, where the interaction between different robots results in more complex and useful behavior. We designed the Limpet to be low-cost and highly manufacturable, which enables us to deploy it in huge collectives for inspecting offshore energy platforms. We show how the Limpet could be used for fault detection in wind turbines, by detecting multiple measurands from a single sensor. This demonstrates one of the possible multi-sensing capabilities of the Limpet. We demonstrate this approach using four different communication systems. We also show how we can do the processing and analysis of the data from the sensor on-board using the microcontroller, which can improve our communication requirements. The signal-processing techniques that we used in this paper are generally applicable to time-variant measurements; however, they must be applied to time-varying, distinct, and systematically reoccurring measurements to augment the sensing capabilities of a robot. We train the classifier offline and transfer its parameters to the Limpet for online classification. During online classification, the Limpet takes an autonomous decision of either transmitting the data or continuing the monitoring process based on whether a fault is detected or not. We apply spectral analysis to the data to classify the type of fault detected. This work shines light on how robots can perform on-board signal processing and analysis to gain multi-functional sensing capabilities, improve their communication requirements, and monitor the structural health of equipment.

## Figures and Tables

**Table 1:** The different physical measurements from each sensor on the Limpet and their relation to different possible measurands.

Sensor	Physical Measurement Variable	Signal Variable	Measurands
Accelerometer	Acceleration	Acceleration	Inclination, Collision, Vibration, Free-Fall Detection, Movement Acceleration
Gyroscope	Angular Velocity	Angular Velocity	Tilt Detection, Orientation
Temperature	Temperature	Temperature	Ambient Temperature, Over-heating, Fire Detection
Humidity	Humidity	Humidity	Relative Humidity
Microphone	Sound	Voltage	Speech Recognition, Noise Cancellation, Audible Fault Detection
Pressure	Pressure	Pressure	Ambient Pressure
Hall-Effect	Magnetic Field	Magnetic Flux Density	Locating Pipelines, Corrosion Detection
Optical	Light (Visible)	Distance	Ambient Light Intensity, Local Communication, Color Detection
Distance (Time-of-Flight)	Light (Laser)	Distance	Fault Detection, Proximity, Collision Detection, Object Identification

## Supplementary Materials

The following are available online at http://www.mdpi.com/1424-8220/18/10/3487/s1.
