# Supplementary material for: The Limpet: A ROS-Enabled Multi-Sensing Platform for the ORCA Hub
Source: Sensors (Basel). 2018 Oct 16;18(10):3487. doi: 10.3390/s18103487 (PMC6210591; doi:10.3390/s18103487)
Supplement: Supplementary file 1 [file sensors-18-03487-s001.zip › Limpet v1.0 User Guide.docx]

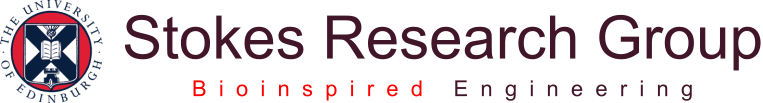


Limpet v1.0

User Guide


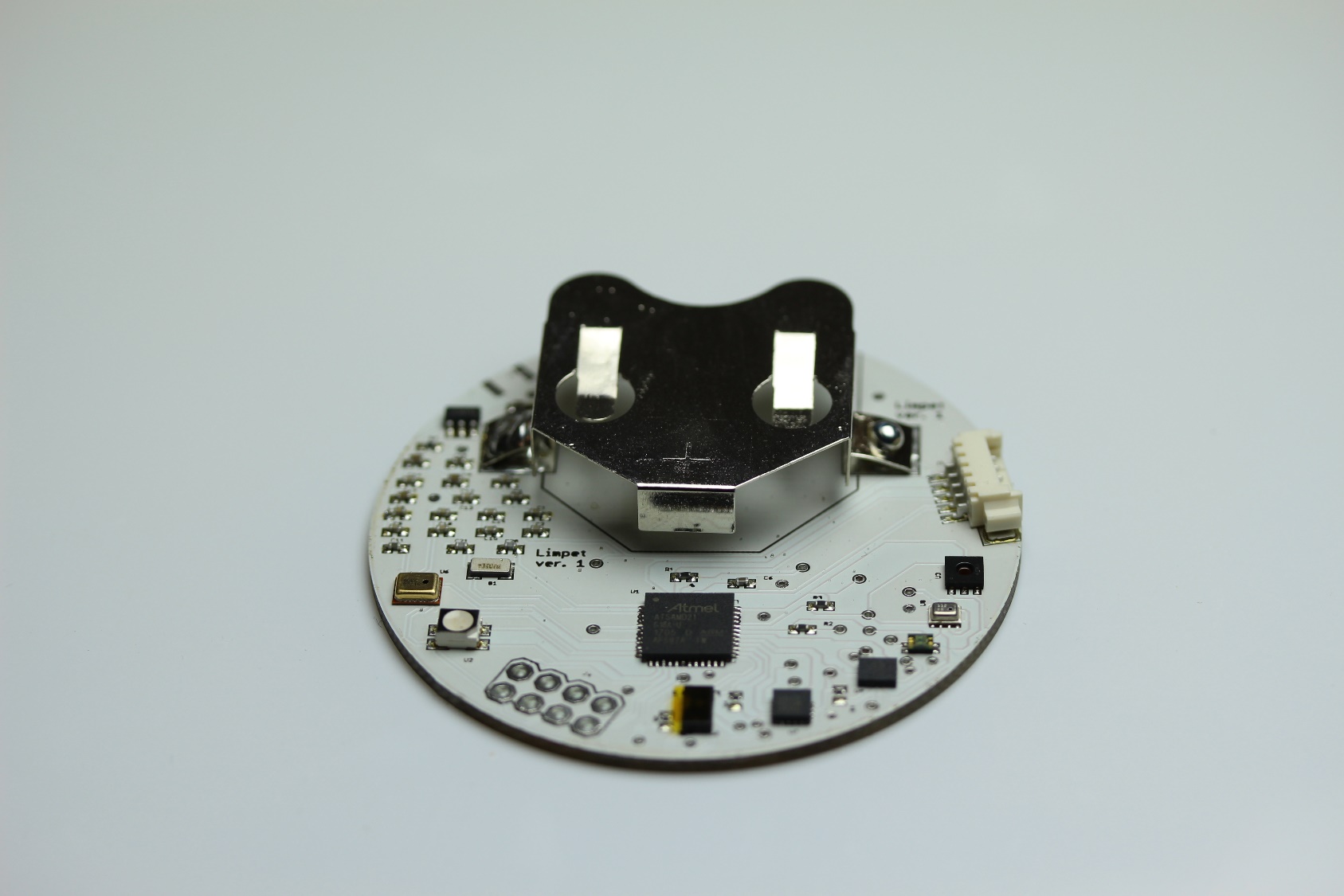


**Mohammed El-Sayed**

Stokes Research Group

The Institute for Integrated Micro and Nano Systems

School of Engineering

The University of Edinburgh

# Introduction


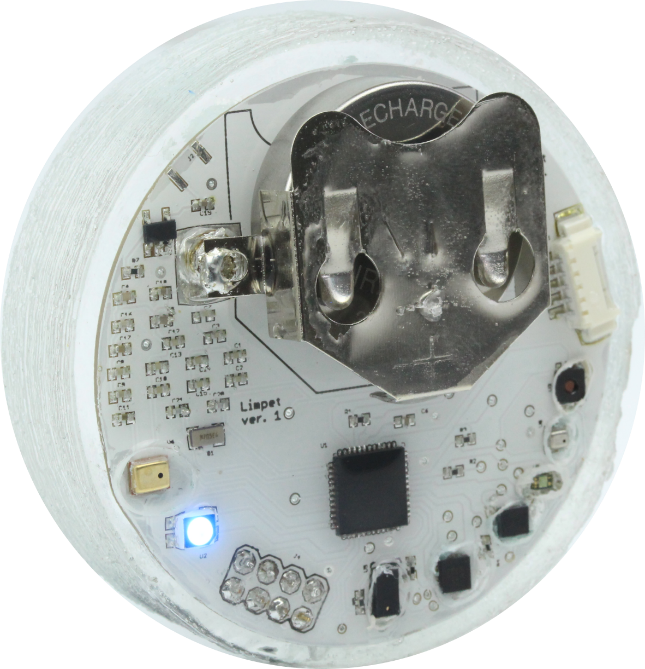
The Limpet is a low cost, miniature and robust robotic platform designed for manufacturability. The Limpet is developed for inspection and monitoring of offshore infrastructures. The Limpet consists of a single two-layer Printed Circuit Board (PCB) and a detachable Li-Ion coin cell battery. The limpet possesses a low-power microcontroller, programming port, exteroceptive sensors and a communication connector. We developed the Limpet for the purpose of inspection and monitoring of offshore energy platforms. It is a multi-sensing platform, equipped with nine exteroceptive sensing modalities, that can be used to monitor a wide range of environmental and physical parameters in offshore platforms. We designed the Limpet as an immobile robot that can attach to metallic surfaces using permanent magnets embedded in its protective housing. The protective housing (polyurethane) acts as a shell protecting the circuitry of the Limpet from the harsh offshore weather conditions. Robust agents will have a longer lifetime, need less maintenance and require lower repair costs. The Limpet is designed explicitly for manufacturability; it consists of a single PCB and therefore mass manufacture is a simple case of placing a batch order with a PCB foundry. The PCB consists of SMD-only components, except for the ESP8266, and can be autonomously populated with pick-and-place machines at the point of manufacture. Assembly of one robot takes seconds as it is a matter of only connecting the coin cell battery. Therefore, it is easy to mass-produce Limpet agents and deploy them in huge collectives for inspecting offshore infrastructures. This guide describes the system’s operation, specifications, properties and usage.

Limpet Schematic


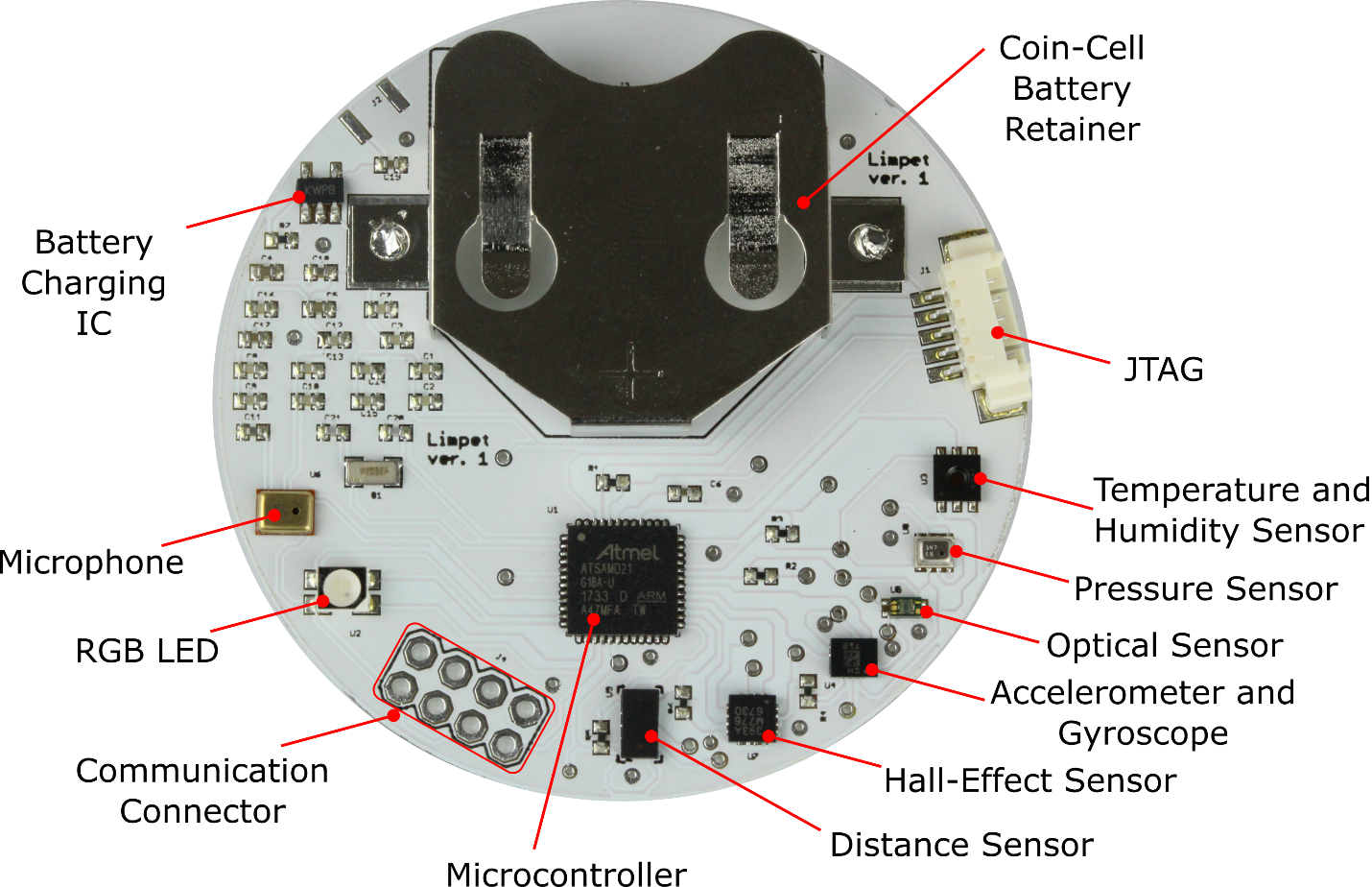


Limpet Board Layout


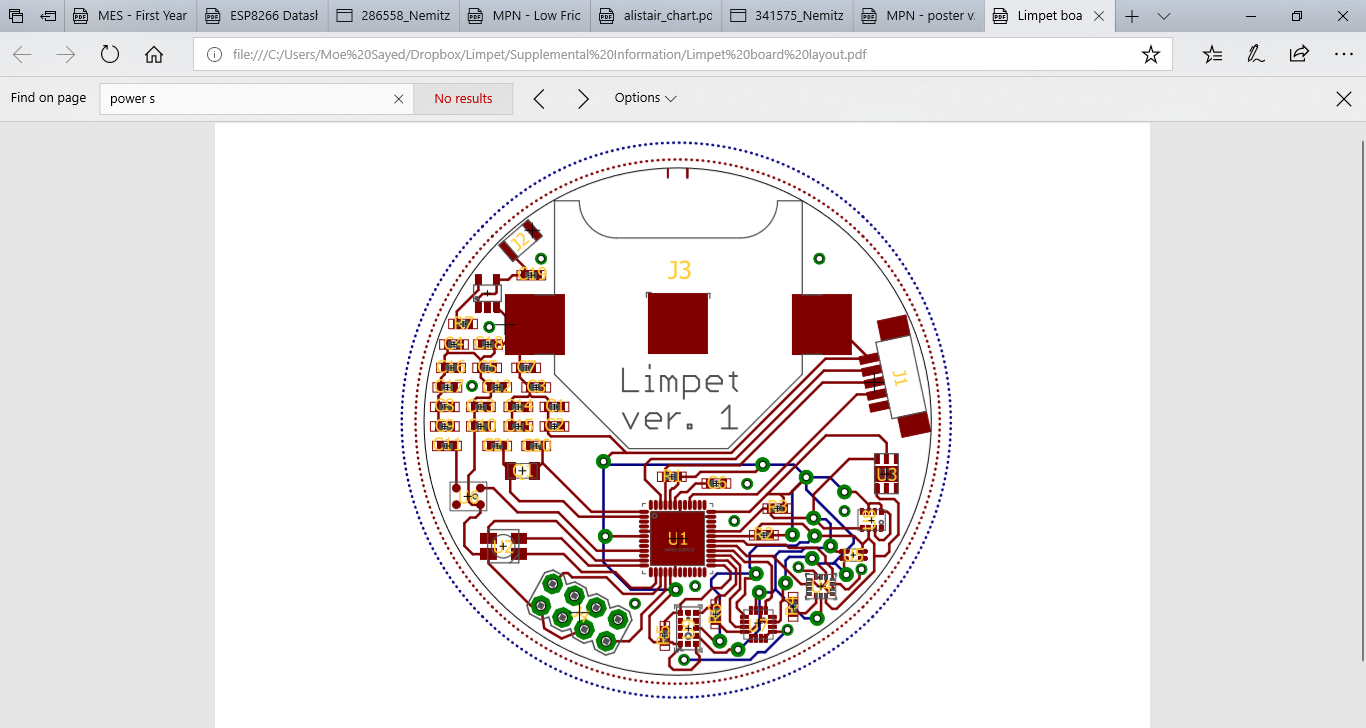


Electrical Specifications

|  | **Min.** | **Typ.** | **Max.** | **Units** |
| --- | --- | --- | --- | --- |
| **Supply Voltage** | 1.62 | 3.3 | 3.63 | V |
| **Supply Current** | - | - | 130 | mA |
| **Current Consumption** | 0.1 | 20 | 48 mA | mA |
| **Storage Temperature** | -40 | 25 | 85 | °C |

Components

The Limpet PCB incorporates a microcontroller (ATSAMD21G18A), RGB LED (LTST-N683EGBW), battery holder (BK-877) for a rechargeable Li-ion battery (LIR2477), charging IC (MCP73812T), charger connector, JTAG connector (Molex 532610571), and an ESP connector to connect a SOC Wi-Fi module (ESP8266). The ESP connector (J4) is connected to the serial port (UART) of the MCU. Therefore, this connector can be used to connect other communication modules, where the picture below demonstrates the pins that can be used to do so. The Limpet PCB includes several exteroceptive sensors, which are:

-
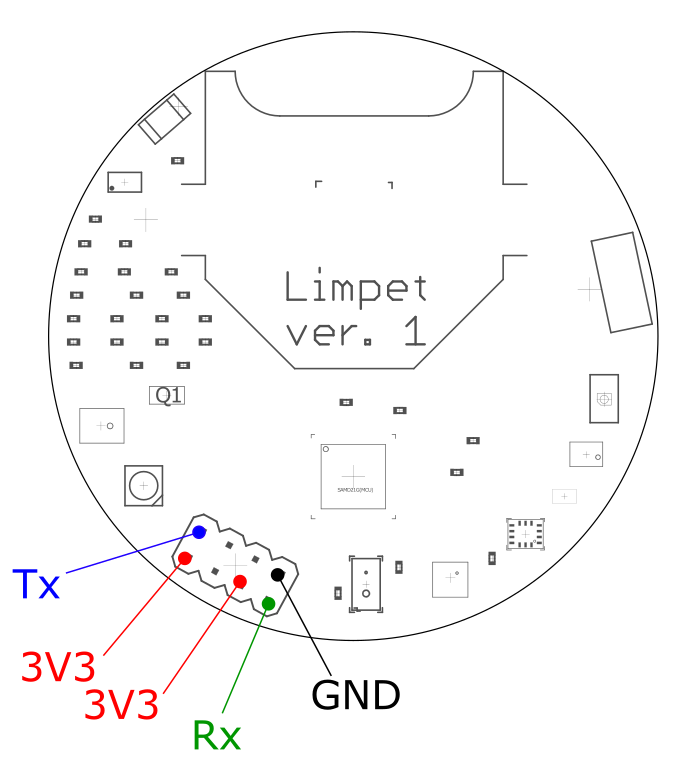
Temperature and Humidity Sensor (Si7006)
- IMU [Accelerometer and Gyroscope] Sensor (LSM6DS3)
- Optical Sensor (VEML6040)
- Sound Sensor (SPU0414HR5H-SB)
- 3-Axis Magnetic Sensor (MLX90393)
- Pressure Sensor (BMP280)
- Distance Sensor (VL53L0)

Sensors

| **Sensor** | **Sensor Modality** | **Measurements** |
| --- | --- | --- |
| **Accelerometer** | Acceleration | - Acceleration (x) - Acceleration (y) - Acceleration (z) - Temperature |
| **Gyroscope** | Angular Velocity | - Angular Velocity (x) - Angular Velocity (y) - Angular Velocity (z) - Temperature |
| **Temperature Sensor** | Temperature | - Temperature |
| **Humidity Sensor** | Humidity | - Relative Humidity |
| **Microphone** | Sound | - Sound Frequency |
| **Pressure Sensor** | Pressure | - Temperature - Pressure - Altitude |
| **Hall-Effect Sensor** | Magnetic Field Strength | - Magnetic Field Strength (x) - Magnetic Field Strength (y) - Magnetic Field Strength (z) - Temperature |
| **Optical Sensor** | Light Frequency | - Correlated Color Temperature - Red Light Power Density - Green Light Power Density - Blue Light Power Density - Ambient Light Intensity |
| **Distance (Time-of-Flight) Sensor** | Laser | - Distance (Range) |

Battery

The Limpet is powered by a 3.6V 160 mAh lithium-ion coin cell battery. The Li-ion battery (LIR2477) is a rechargeable battery and has a diameter of 24.5 mm. The battery can be recharged by using a 6Vdc plug-in power supply. The limpet includes header pins (J2) on the PCB for connecting the power supply to recharge the battery. When the charging IC receives 6V dc, it will start charging the onboard battery until the battery is full.

Lifetime

The Limpet has a battery life @160 mAh of 0.87 to 1600 hours. The minimum battery life is calculated by assuming the Limpet has all the sensors, RGB LED, MCU and communication constantly on. The Limpet will consume approximately 182.9 mA (ESP8266 consumes 135 mA, RGB LED consumes 20 mA, sensors consume 20.9 mA, MCU consumes 7 mA) which allows for a battery life time of about 0.87 hours or 52.2 minutes. The maximum battery life is calculated by assuming the Limpet is in sleep mode, where it consumes an average current of 0.1 mA. In this mode, the battery life time of the Limpet can reach about 1600 hours or 67 days.

Cost and Dimensions

The Limpet has a diameter of 50 mm, a height of 7 mm and weighs 17g with, and 10g without, the battery. The total cost of electronic components used in the limpet design is about £22. The cost of the ESP8266 module is £5.

Adhesion Mechanism

Limpet v1 incorporates permanent magnets in the bottom layer for adhesion to metal surfaces. Limpet v2 will include an adhesion mechanism to attach to multiple different surfaces.

Primary Communication Method

The Limpet uses Wi-Fi to communicate and send data to the PC. It incorporates the ESP8266, which is a SOC Wifi module with integrated TCP/IP protocol stack capable of giving any microcontroller access to the WiFi network. The ESP8266 sends the data wirelessly to the PC using a messaging protocol known as MQTT. MQTT is a light-weight publish/subscribe messaging protocol used for remote communication. The received data can then be plotted in a real-time basis using Matlab, or saved and processed later.

Other Communication Methods

The Limpet was designed to have robust communication. It can use one of multiple communication methods, including *Serial*, *WiFi*, *LoRa, Optical* and *Acoustic Communication*. These communication methods have been successfully tested on the Limpet. This gives the Limpet robustness in communication because if the primary communication method (WiFi) fails for any reason, secondary communication methods are available to achieve communication fail-over.

WiFi and serial communication do not allow for robot-to-robot communication. LoRaWAN, Acoustic and optical communication allow for robot-to-robot communication. Optical communication is achieved with a combination of the RGB LED and optical sensor. The messages are encoded in the LED signal by pulse-width modulating the LED to provide a range of different intensities. Each intensity corresponds to a different number from 0 to 9. The different LED colors can be used to correspond to different measurements such as time, distance, pressure, temperature, etc. The optical sensor can measure the red, blue and green light power density as well as the ambient light intensity. The optical sensor can thus use the light intensity and color power density measurements to infer transmitted messages. LoRaWAN is a digital wireless data communication technology that enables long-range transmission with low power consumption. LoRaWAN technology is provided by the LoRa Alliance, which is a non-profit association of more than 500 member companies that are developing and promoting LoRaWAN open standard for IoT.

Setting up the ESP8266

Please refer to the ESP8266 tutorial for information on how to setup the ESP8266.

Programming the Limpet


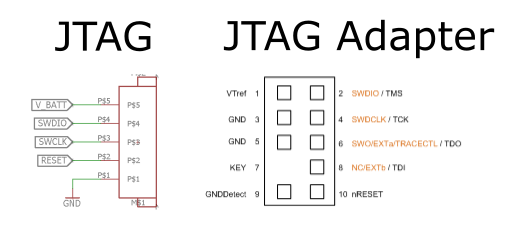
The limpet has an onboard programming port (JTAG). The limpet is programmed using a J-Link programmer together with a JTAG Adapter (Olimex ARM-JTAG-20-10). The limpet is programmed in C/C++ using Atmel Studio 7. The connection between the JTAG Adapter and the programming port (JTAG) is shown below.

Data Treatment on the Limpet

In data treatment, there are two distinguished phases: data processing and data analysis. Data processing includes data visualization, eliminating outliers, averaging, smoothing, down sampling, quality check, etc. This phase aims to remove noise and outliers from the data to prepare it for further processing and analysis. The optimum data product is the collection of information that best represents the measured parameter and that requires the minimum manipulation to be embedded in a particular analysis.

The Limpet allows for on-board processing and analysis of data collected from the various sensors. The on-board processes can eliminate any noise and outliers, smooth the data, down-sample the data to accord with computational limitations of the microcontroller, and check the data against pre-stored values. These processing capabilities are just a few of the on-board processing techniques that can be carried out on the Limpet. The Limpet can use these processing capabilities to make autonomous decisions during inspection and monitoring of offshore energy platforms. The user can remove or add new data processing stages if required. The choice of data treatment ultimately depends on the nature of the monitored variable, the final usage of the data, the environmental conditions, the monitoring setup, power consumption and communication method. Each use case can have its own customized set of processing on the Limpet. The Limpet can also perform multiple on-board analysis processes such as: Spectral Analysis, Acoustic Fingerprinting, Sliding Window Analysis, and many others.

ROS Interface

The Limpet can be integrated with ROS interface. The figure below shows an overview of the ROS interface with the Limpet. The sensor data is fed into the microcontroller. The microcontroller sends out the data to a converter, which converts this data into a ROS protocol. The data can be published to a ROS topic and any ROS node can subscribe to that topic to read the sensor data. The different sensors on the Limpet have different physical measurement variables. These measurement variables are fed into the microcontroller, where it adds a label to the data to differentiate data from the different sensors (e.g. distance, temperature, pressure, etc). The microcontroller sends out the data to a converter, which converts this data into a ROS protocol. The data can be published to a ROS topic and based on the label sent with the data, the converter can decide which topic to publish the data to. ROS nodes can subscribe to the different topics to gain access to the sensor data. This enables the Limpet to be used as part of a multi-robotic system, where the interaction between different robots results in a more complex and useful behavior. For example, Limpets can be deployed from onshore to offshore platforms using drones, and they can then be repositioned using the drones to create a dynamic multi-
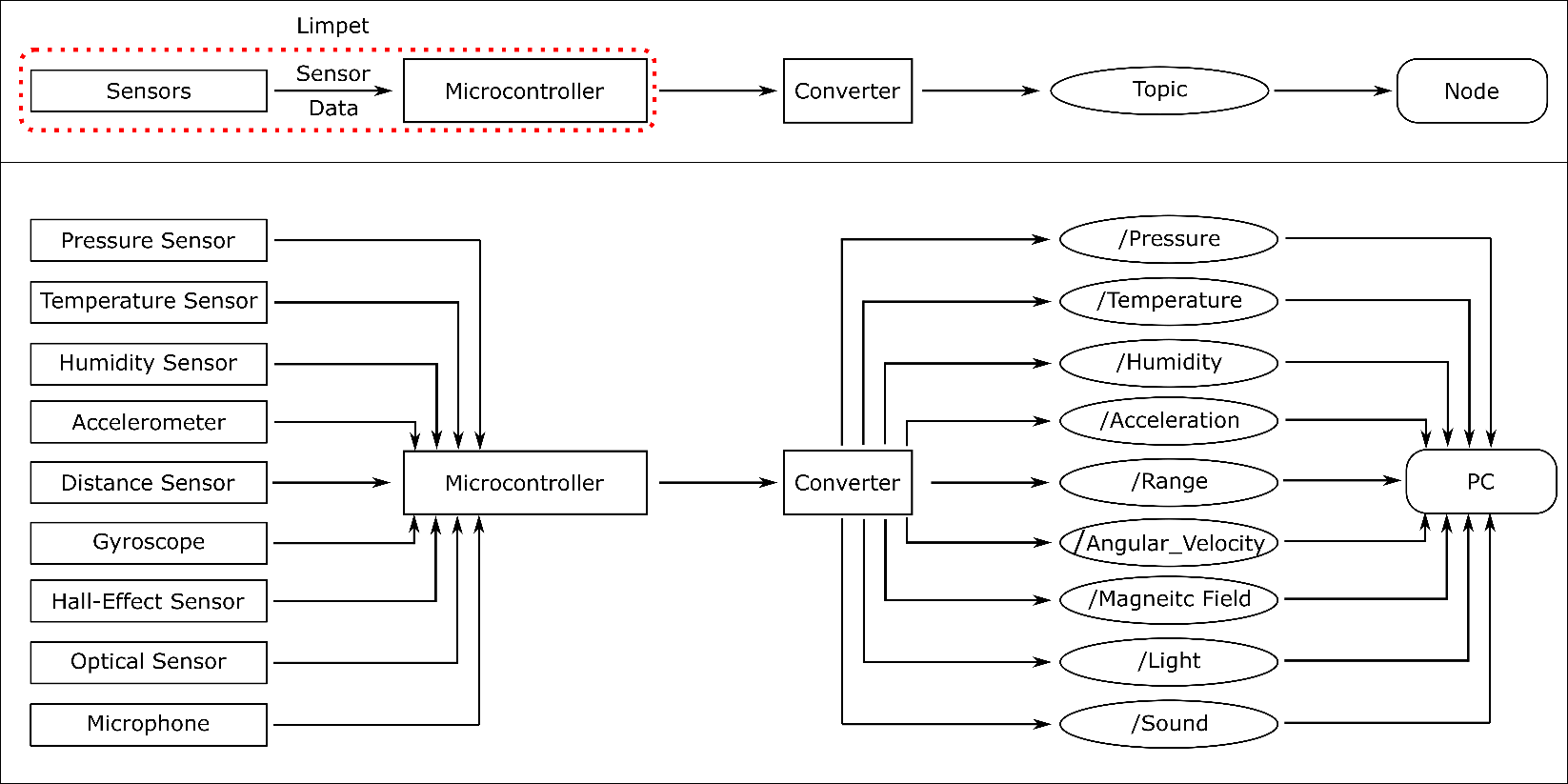
sensing robotic system.
