# Supplementary material for: The Limpet: A ROS-Enabled Multi-Sensing Platform for the ORCA Hub
Source: Sensors (Basel). 2018 Oct 16;18(10):3487. doi: 10.3390/s18103487 (PMC6210591; doi:10.3390/s18103487)
Supplement: Supplementary file 1 [file sensors-18-03487-s001.zip › Limpet Supplemental Information.docx]

The Limpet: A ROS-Enabled Multi-Sensing Platform for the ORCA Hub

Mohammed E. Sayed ^1^, Markus P. Nemitz ^1,†^, Simona Aracri ^1,†^, Alistair McConnell ^1^, Ross M. McKenzie ^1,2^ and Adam A. Stokes ^1,*^

**SUPPLEMENTAL INFORMATION**

^1^School of Engineering, Institute for Integrated Micro and Nano Systems, The University of Edinburgh, Scottish Microelectronics Centre, Alexander Crum Brown Road, King's Buildings, Edinburgh, UK , EH9 3FF

^2^Engineering and Physical Sciences Research Council (EPSRC) Centre for Doctoral Training (CDT) in Robotics and Autonomous Systems, School of Informatics, The University of Edinburgh, Edinburgh EH9 3LJ, UK

*** Author to whom correspondence should be addressed.**

† These authors contributed equally to this work.

# Supplemental Figures


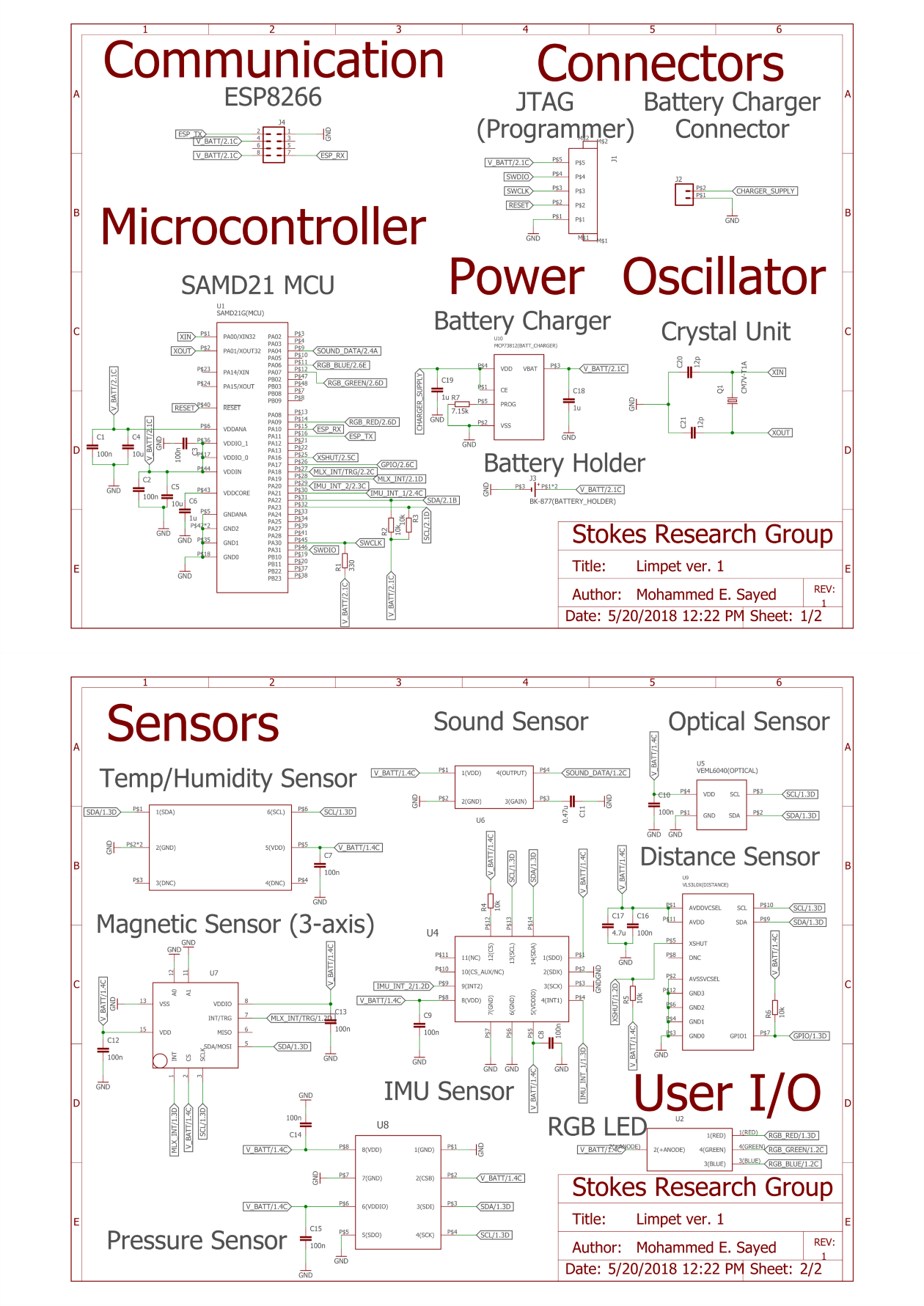


Figure S1: PCB schematic of the Limpet.


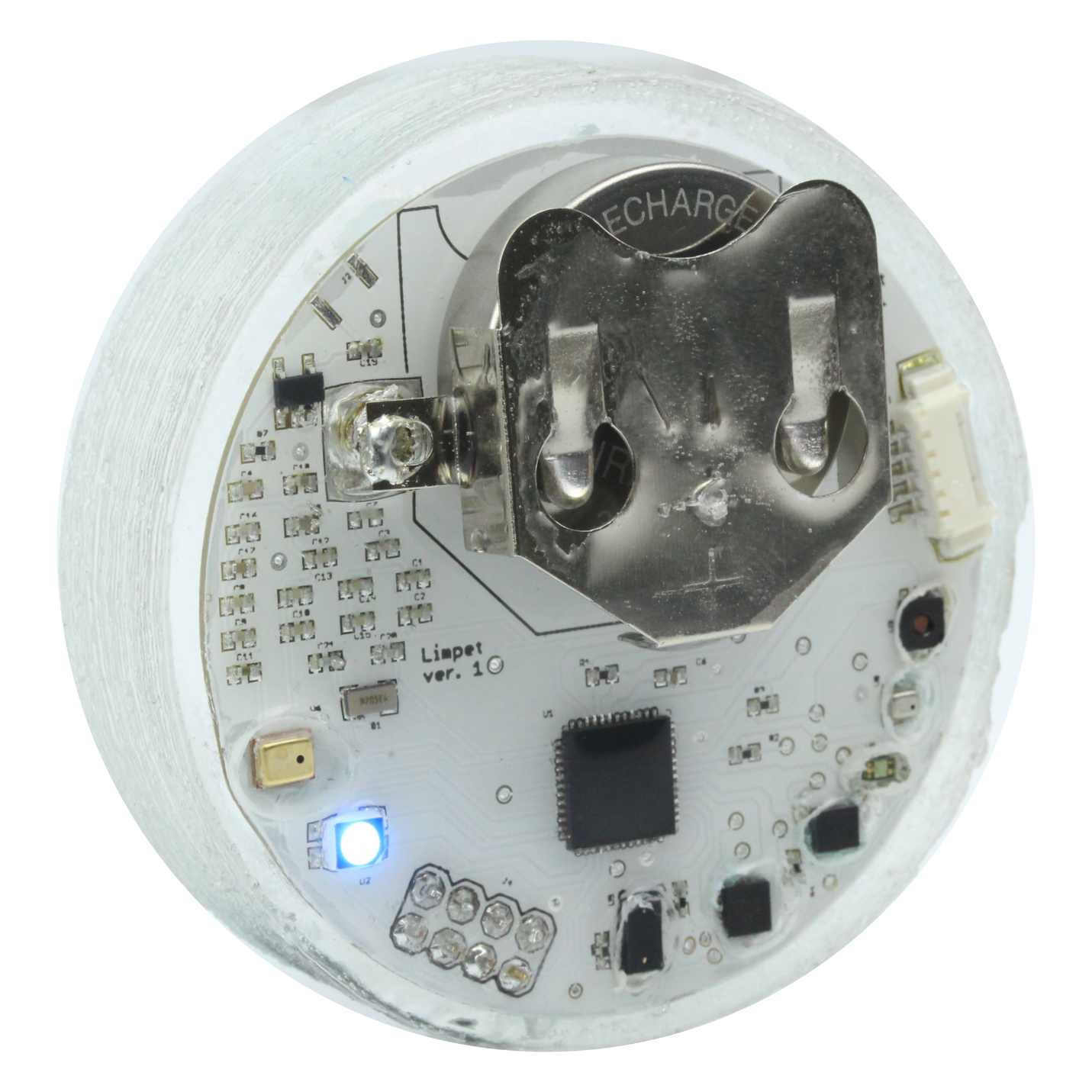


Figure S2: A picture of a Limpet in a protective housing made of clear polyurethane resin.


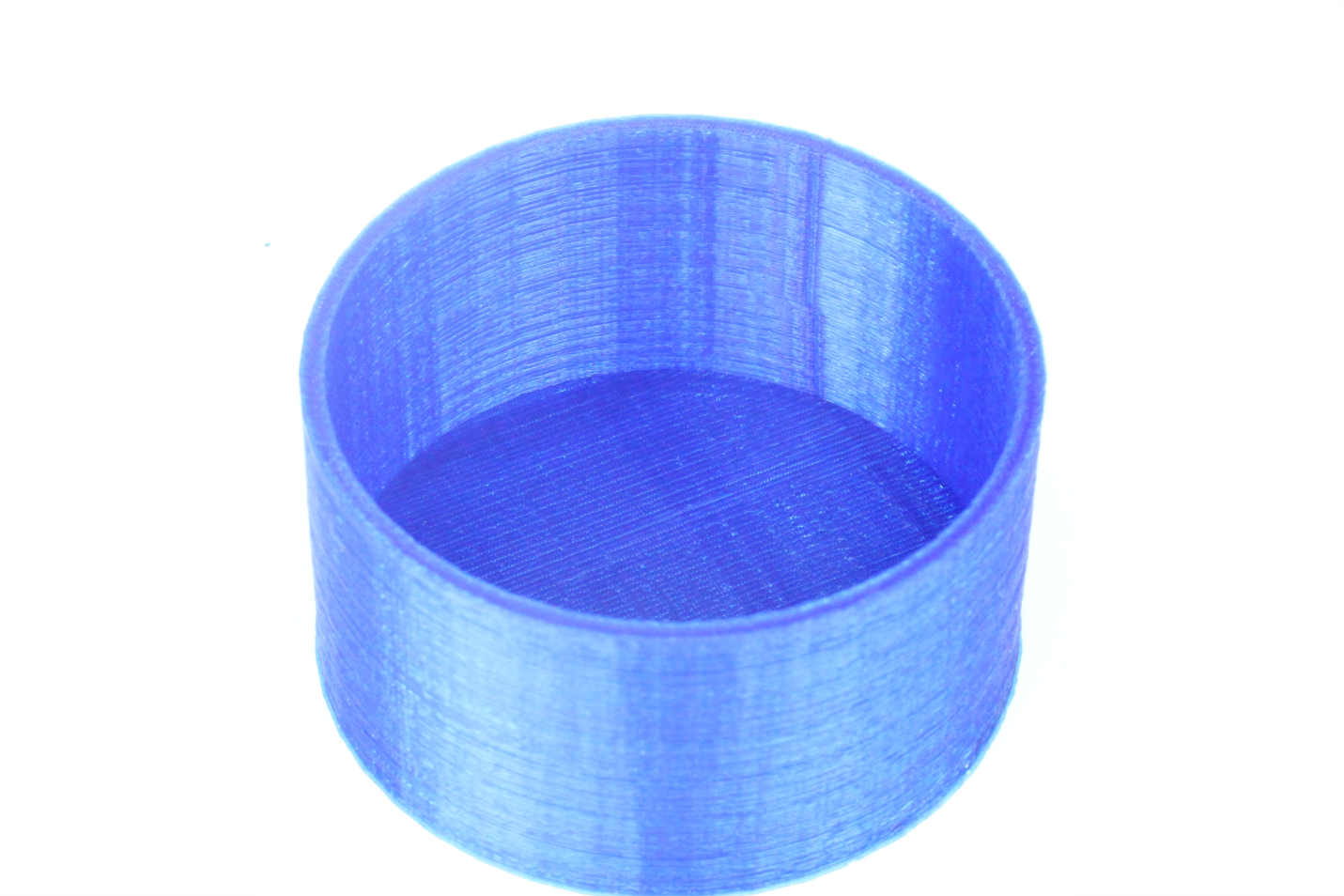


Figure S3: A picture of the 3D printed mold used to design the protective housing of the Limpet.


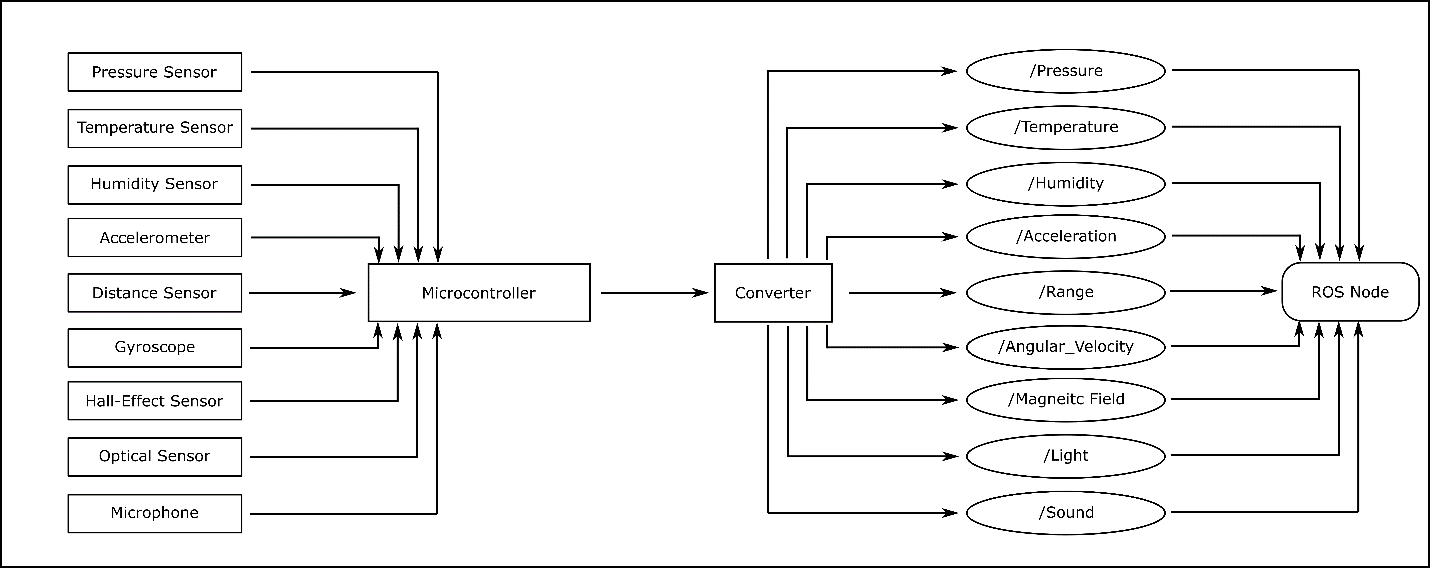


Figure S4: An overview of the ROS Interface with the Limpet. The different sensors on the Limpet have different physical measurement variables. These measurement variables are fed into the microcontroller, where it adds a label to the data to differentiate data from the different sensors (e.g. distance, temperature, pressure, etc). The microcontroller sends out the data to a converter, which converts this data into a ROS protocol. The data can be published to a ROS topic and based on the label sent with the data, the converter can decide which topic to publish the data to. Any ROS node can subscribe to a specific ROS topic to read the sensor data.


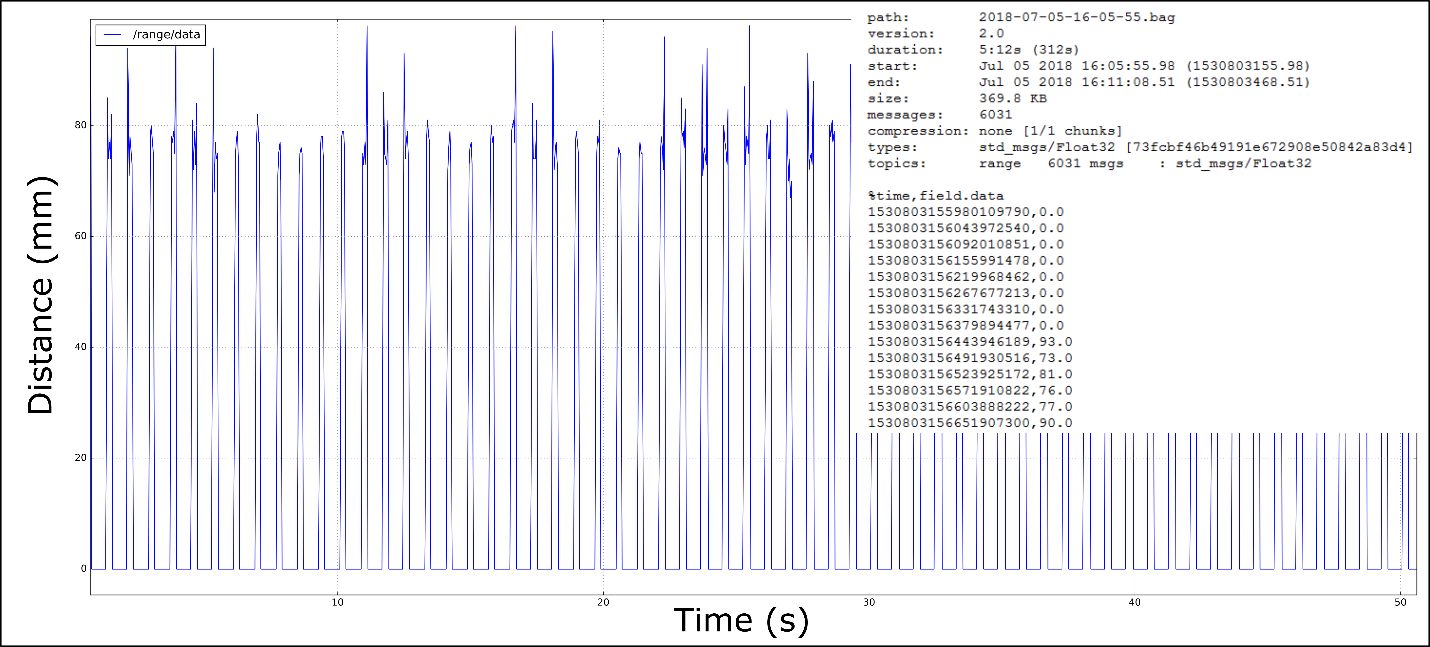


Figure S5: A figure showing a screenshot of the data published to the distance ROS Topic, and a graph of the distance measurement from the ROS Topic during normal operation of the fan.


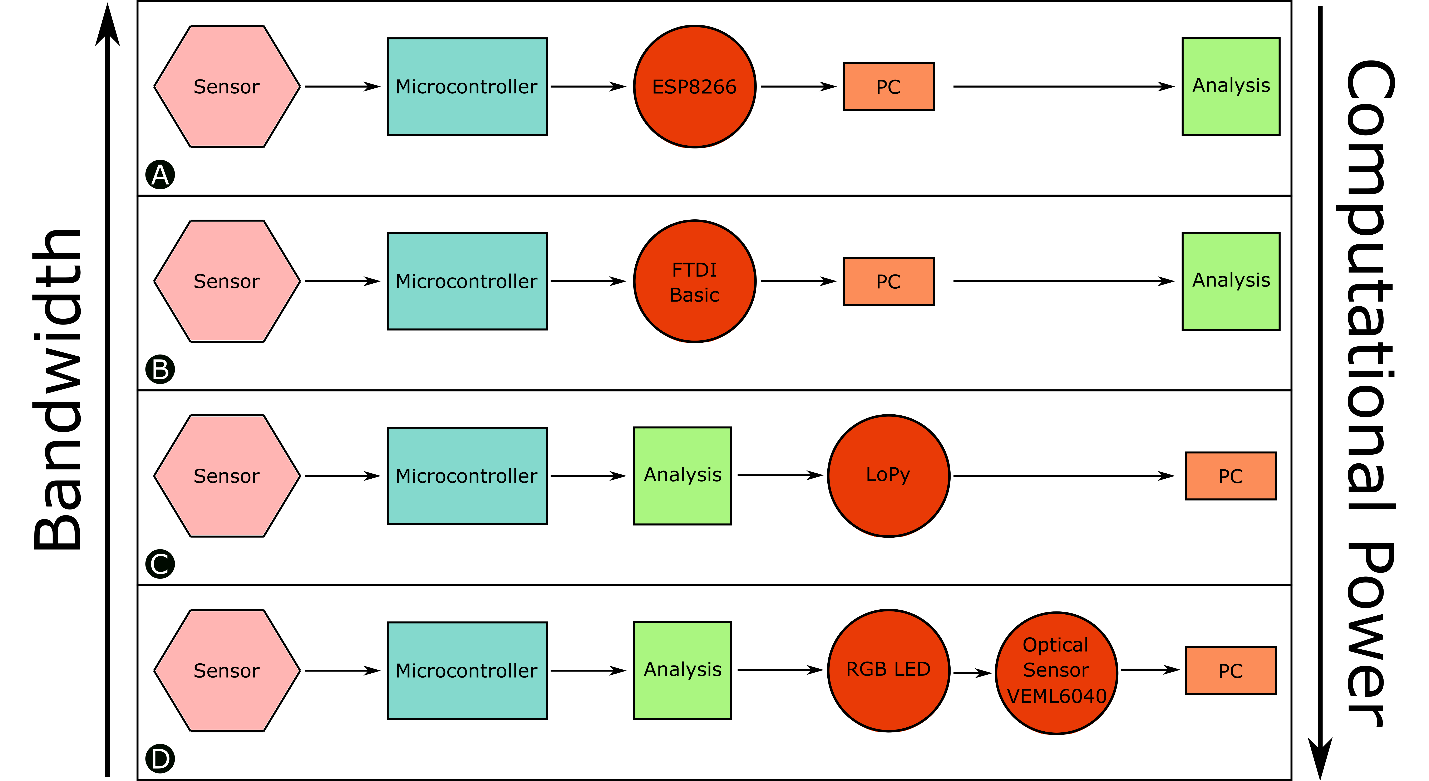


Figure S6: Overview of the communication strategies. The communication bandwidth decreases and the on-board computational power increases as we move from WiFi to optical communication. A) Overview of components involved in WiFi communication. In this communication strategy, the sensor data is fed into the microcontroller, which is then sent in real time to the PC. Transmission of data over WiFi is achieved using the ESP8266, a SOC WiFi module with integrated TCP/IP protocol stack that gives the Limpet access to the WiFi network. The data can then be analysed on the PC. B) Overview of components involved in serial communication. Similar to WiFi communication, the sensor data is fed into the microcontroller, which is then sent in real time to the PC. Transmission of data using serial communication is achieved using SparkFun’s FTDI Basic Breakout - 3.3V. The FTDI Basic is a serial to USB converter. The Limpet transmits the data through the FTDI Basic, which can be recorded on the PC using a terminal program and analysed later. C) Overview of components involved in LoRa communication. LoRa has a much lower communication bandwidth than serial and WiFi communication, thus, processing and analysis need to be done before transmission to allow for only small packets of data to be transmitted. This on-board processing and analysis requires higher computational power than serial and WiFi communication. To transmit data via LoRa, we used the LoPy, which is a MicroPython enabled WiFi, Bluetooth and LoRa development board. D) Overview of components involved in optical communication. Optical communication has the lowest bandwidth in these communication strategies. The processing and analysis also need to be done before the transmission of data. In this case, the transmission of data was achieved using the on-board RGB LED and the optical sensor. The data from the LED are transmitted by pulse-width modulating the LED to different levels. The optical sensor was used to read the light intensity and colour power density to interpret the data transmitted using the RGB LED.

**
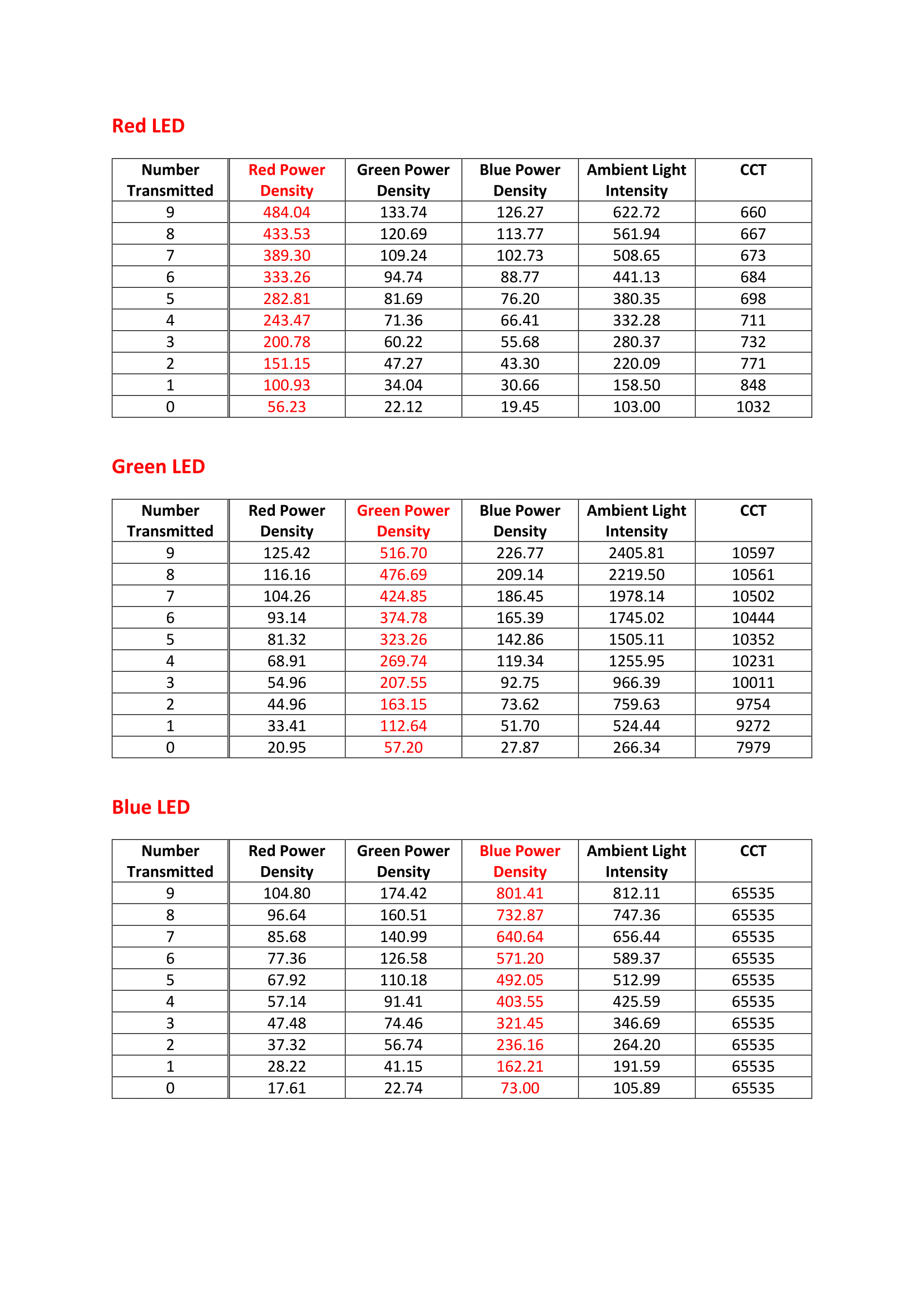
**

Figure S7: A figure showing the power density corresponding to the number transmitted by the red (Table 1), green (Table 2) and blue (Table 3) LEDs. These measurements were used as a reference to interpret the numbers transmitted by the Limpet during the experiments.


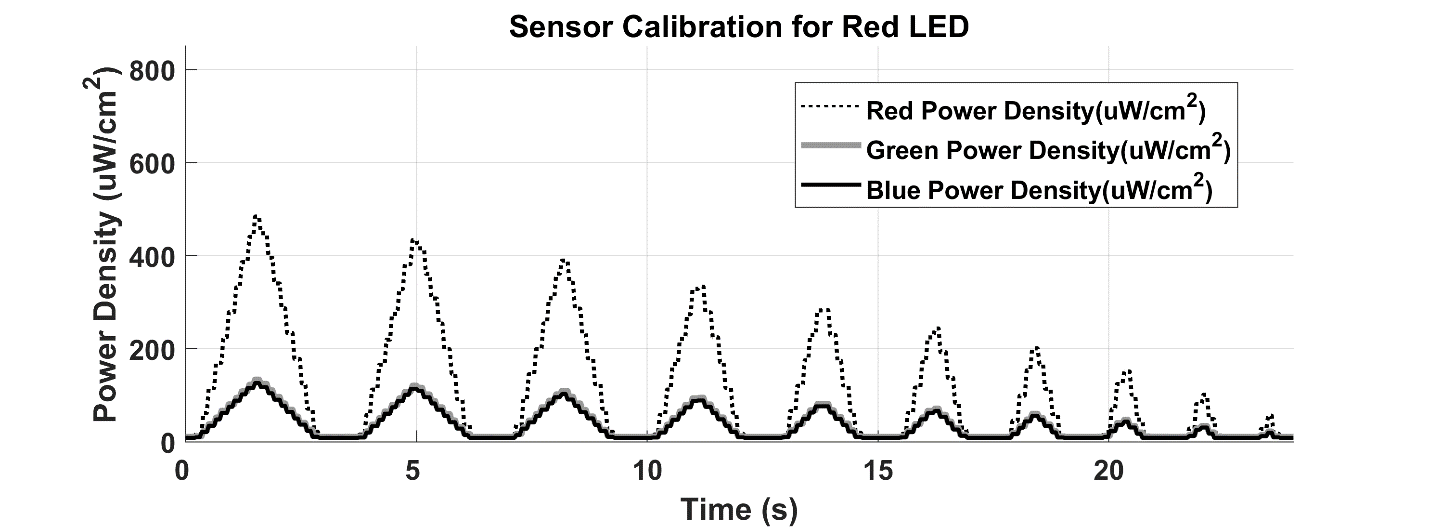


Figure S8: A plot of the power density data versus time from the optical sensor when transmitting numbers using the red LED. Each consecutive peak corresponds to a number from 9 to 0.


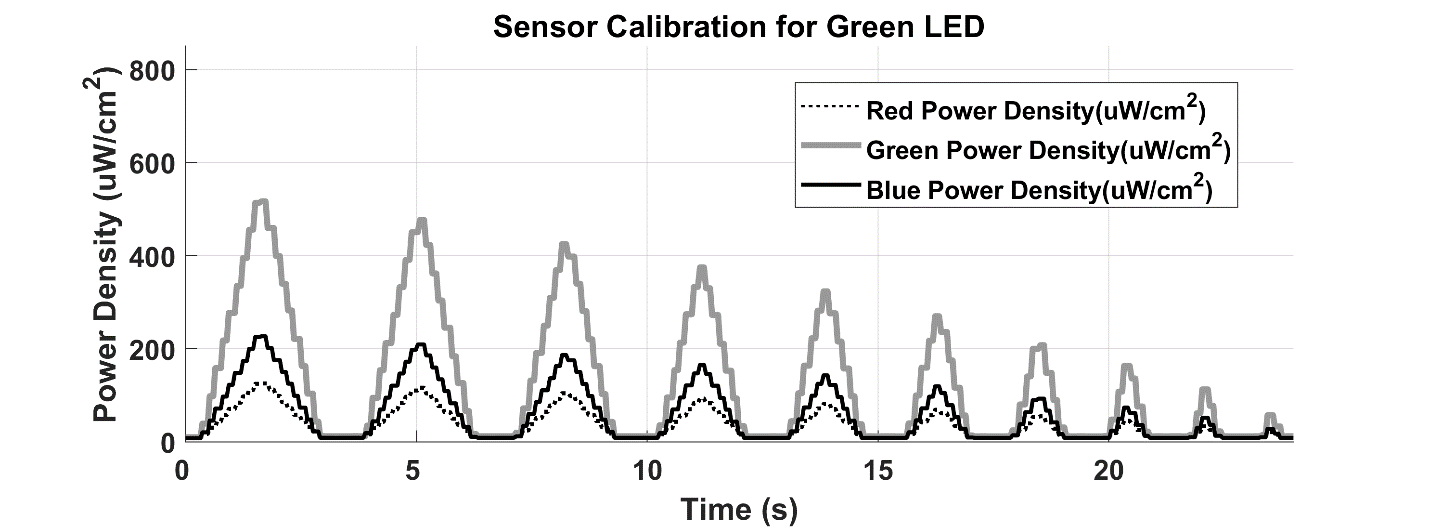


Figure S9: A plot of the power density data versus time from the optical sensor when transmitting using the green LED. Each consecutive peak corresponds to a number from 9 to 0.


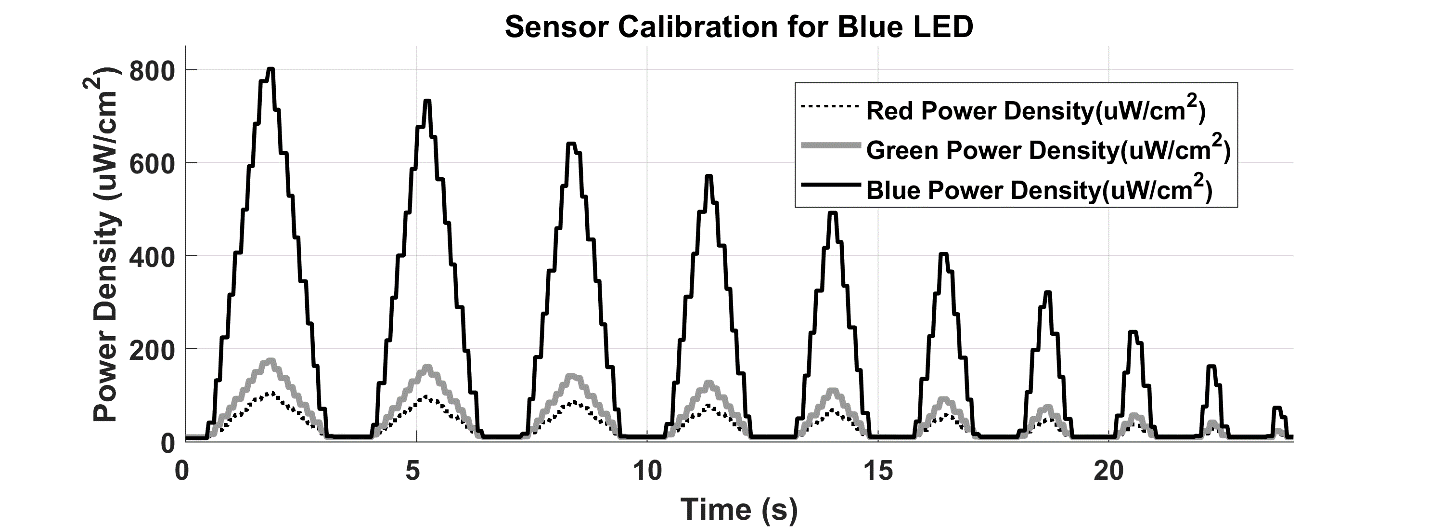


Figure S10: A plot of the power density data versus time from the optical sensor when transmitting numbers using the blue LED. Each consecutive peak corresponds to a number from 9 to 0.


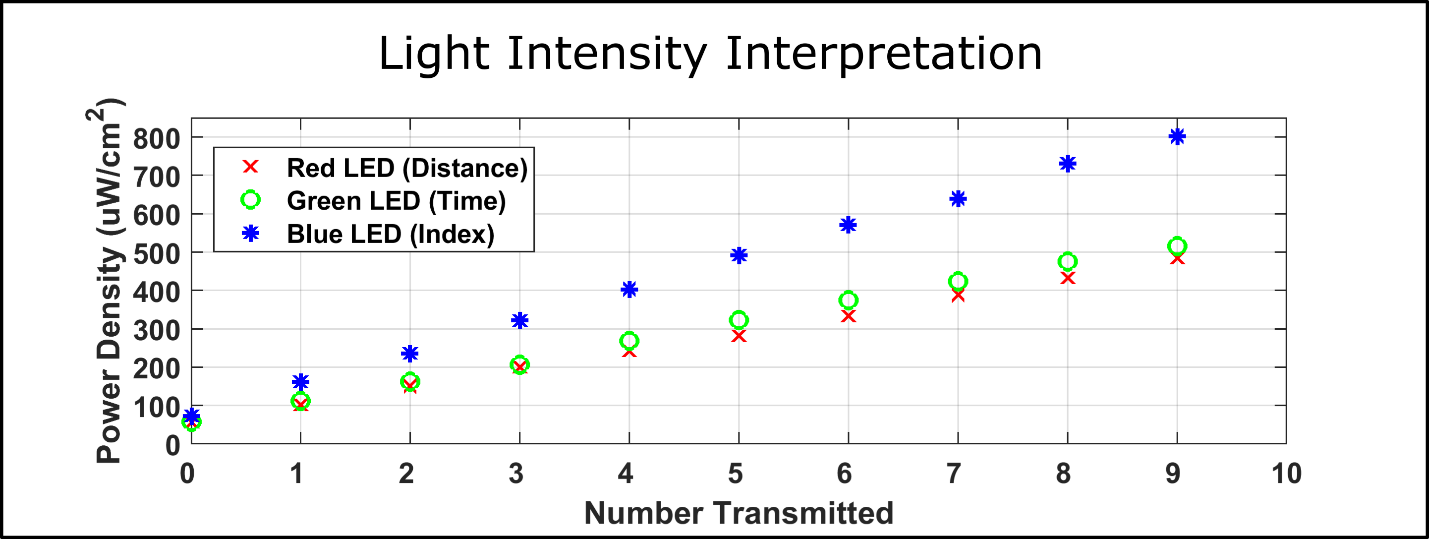


Figure S11: Light intensity interpretation for optical communication. This graph shows how the power density of the RGB LED represents a number transmitted by the Limpet. The red LED is used to send information about distance, green LED is used for timestamp information and blue LED is used for index information. The different power density for each LED corresponds to a number on a scale of 0 to 9. The different power densities are achieved by pulse-width modulating the RGB LED to different levels.


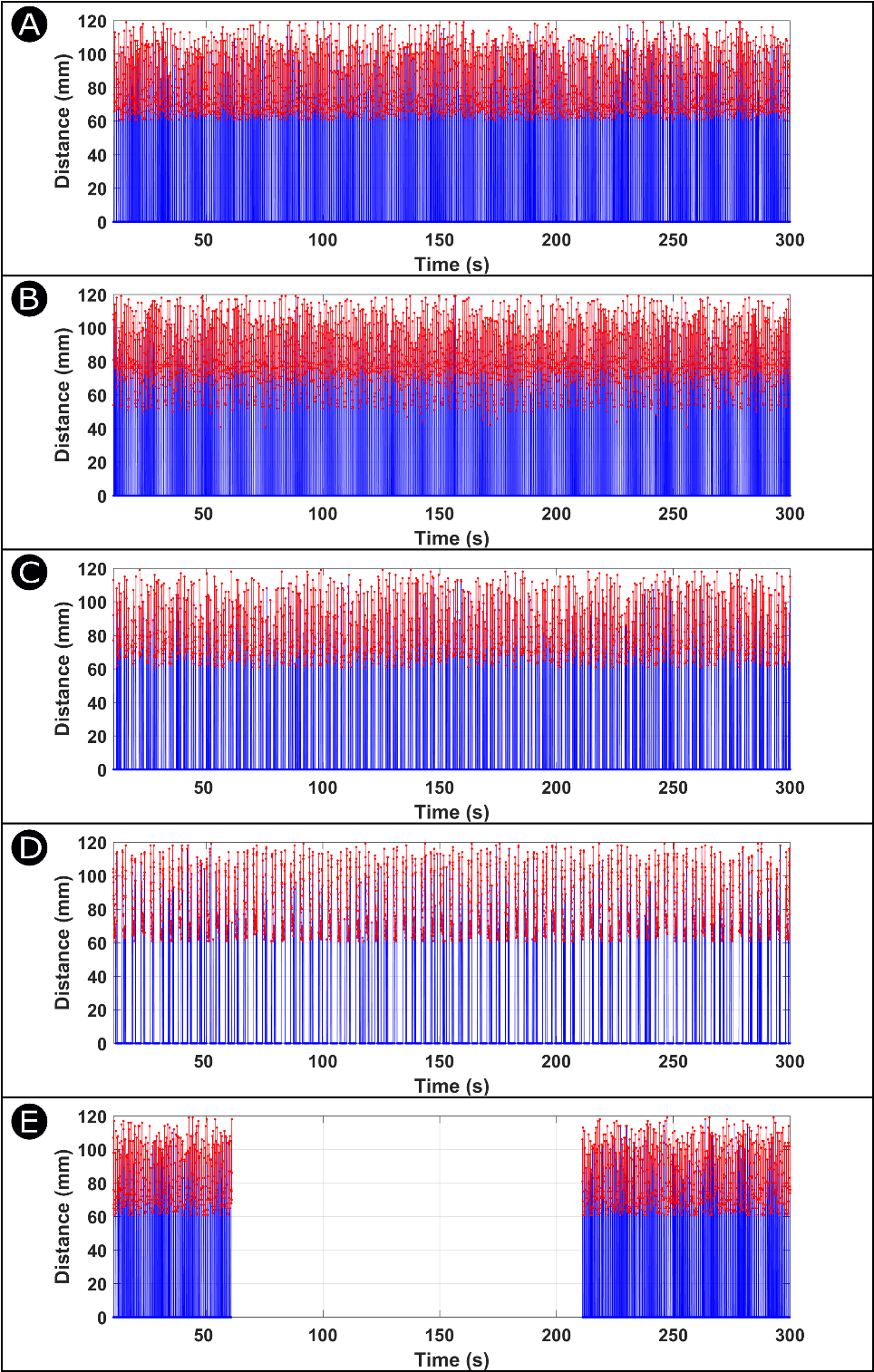


Figure S12: A figure showing the raw sensor data transmitted by the Limpet via WiFi during A) normal operation, B) Fault 1, C) Fault 2, D) Fault 3, E) Fault 4. The sensor data has been recorded over a period of 300 seconds.


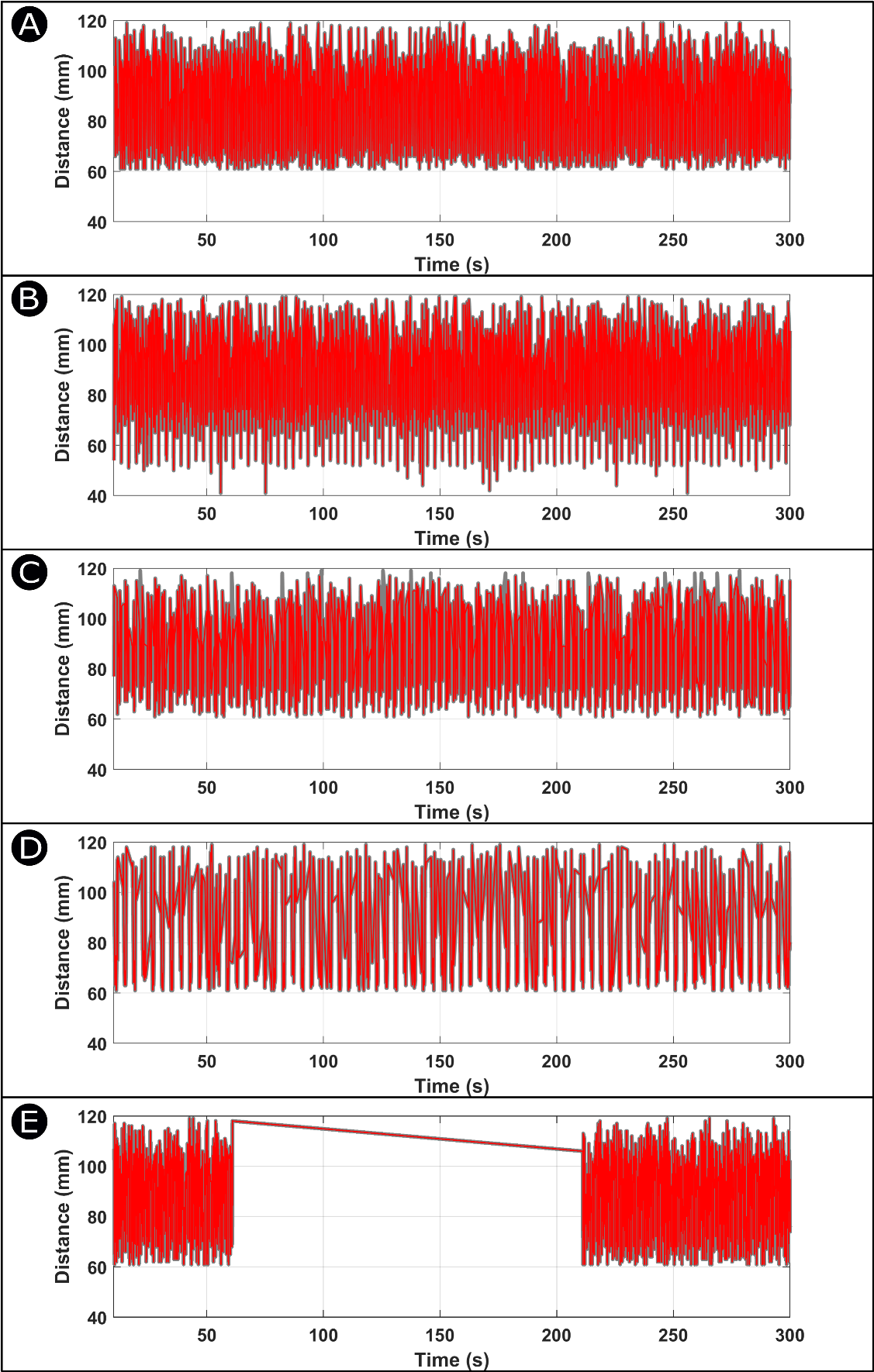


Figure S13: A figure showing the sensor data, which was transmitted via WiFi, after elimination of outliers during A) normal operation, B) Fault 1, C) Fault 2, D) Fault 3, E) Fault 4. The sensor data has been recorded over a period of 300 seconds.


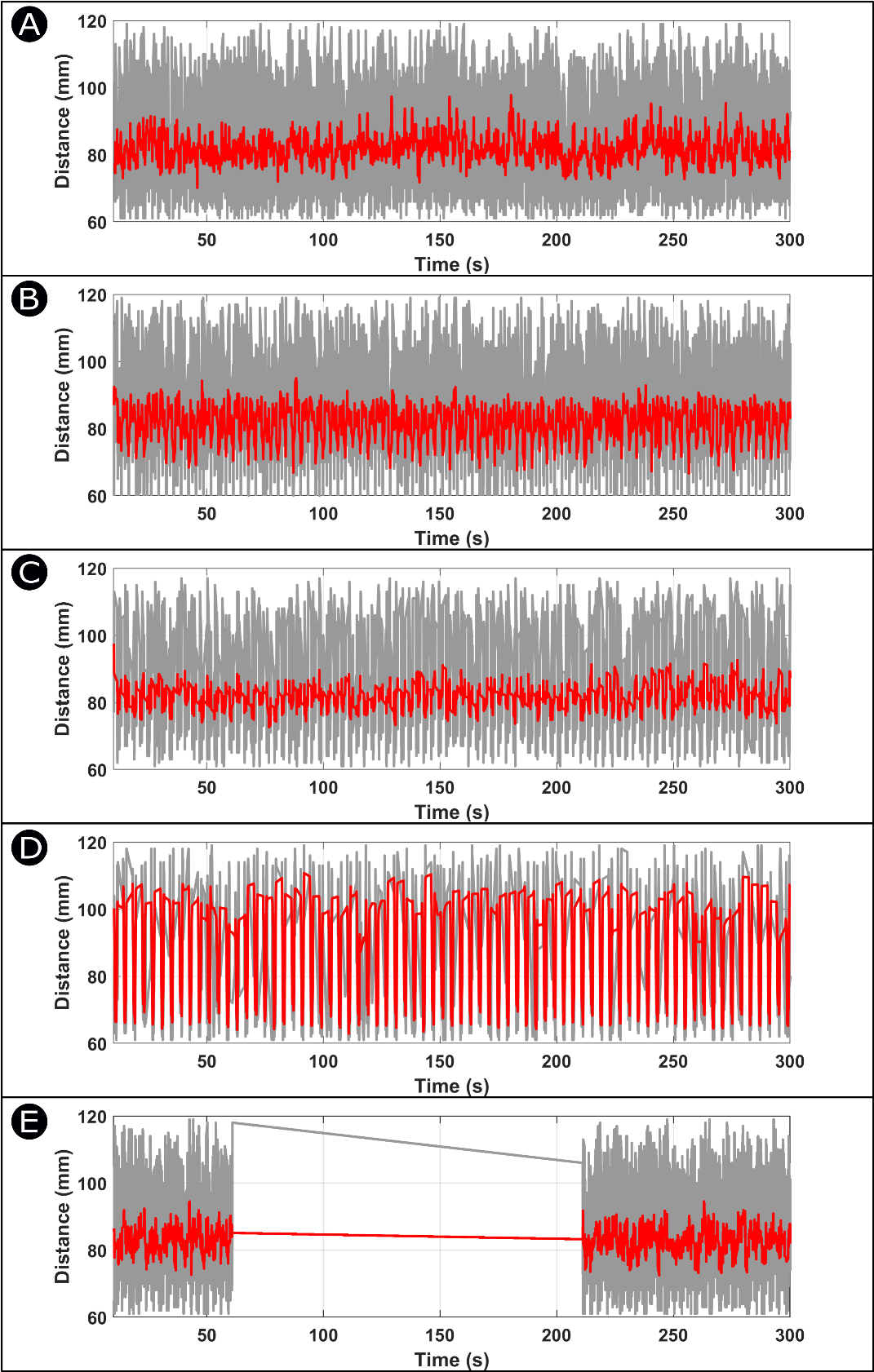


Figure S14: A figure showing the sensor data, which was transmitted via WiFi, after application of a low-pass filter during A) normal operation, B) Fault 1, C) Fault 2, D) Fault 3, E) Fault 4. The sensor data has been recorded over a period of 300 seconds.


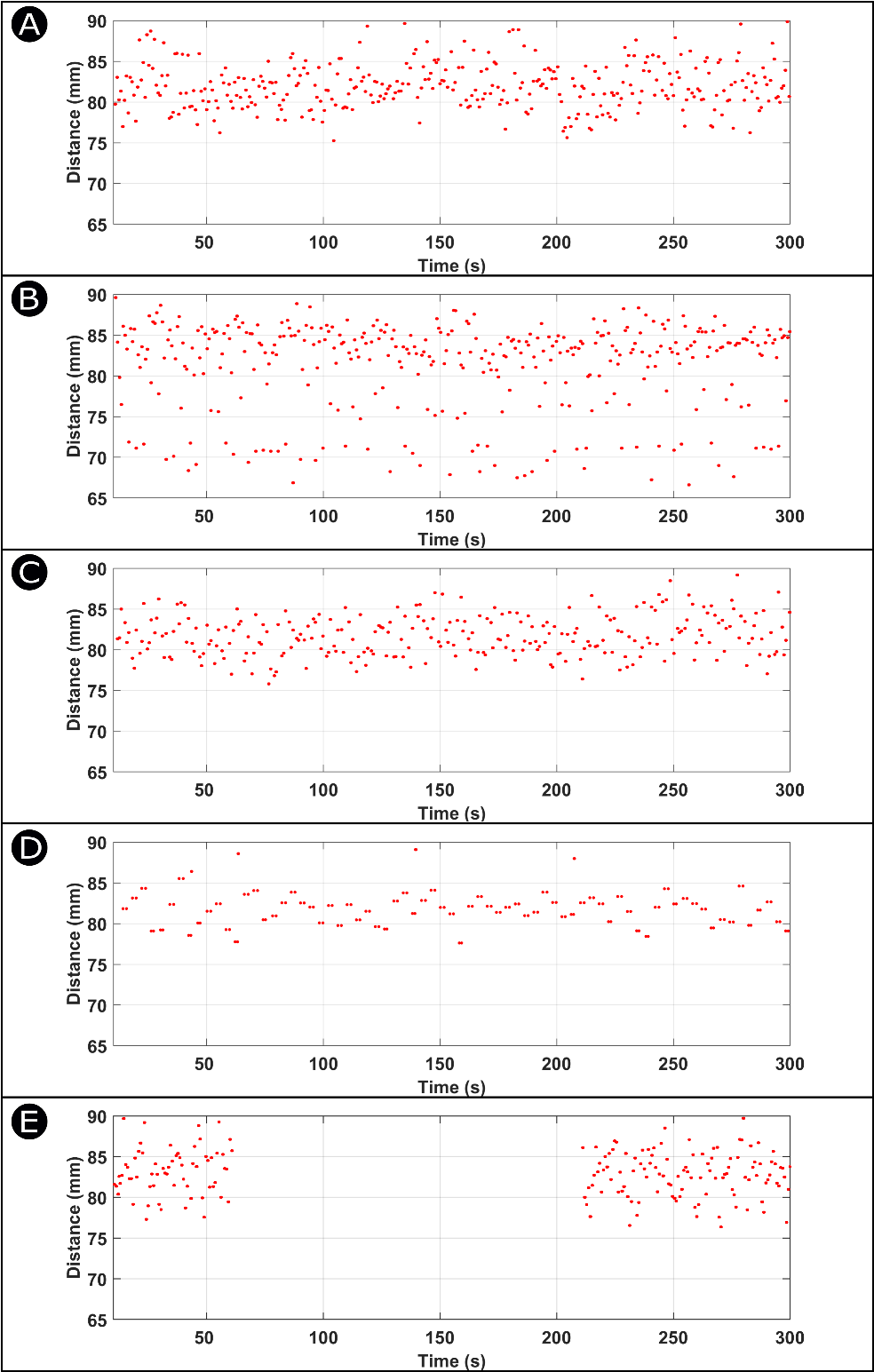


Figure S15: Down-sampling results from WiFi communication. This figure shows the effect of down-sampling on the sensor data, which was transmitted via WiFi, after removing the outliers and applying the low-pass filter. The sensor data was recorded over a period of 300 seconds. Our down-sampling method decreases a signal’s length to a number of averaged data points representing the fan blades. A) This graph shows the effect of down-sampling on the sensor data for normal operation mode. B) This graph shows the effect of down-sampling on the sensor data for fault 1 operation mode. C) This graph shows down-sampled data for fault 2 operation mode. D) This graph shows down-sampled data for fault 3 operation mode. E) This graph down-sampled data for fault 4 operation mode.


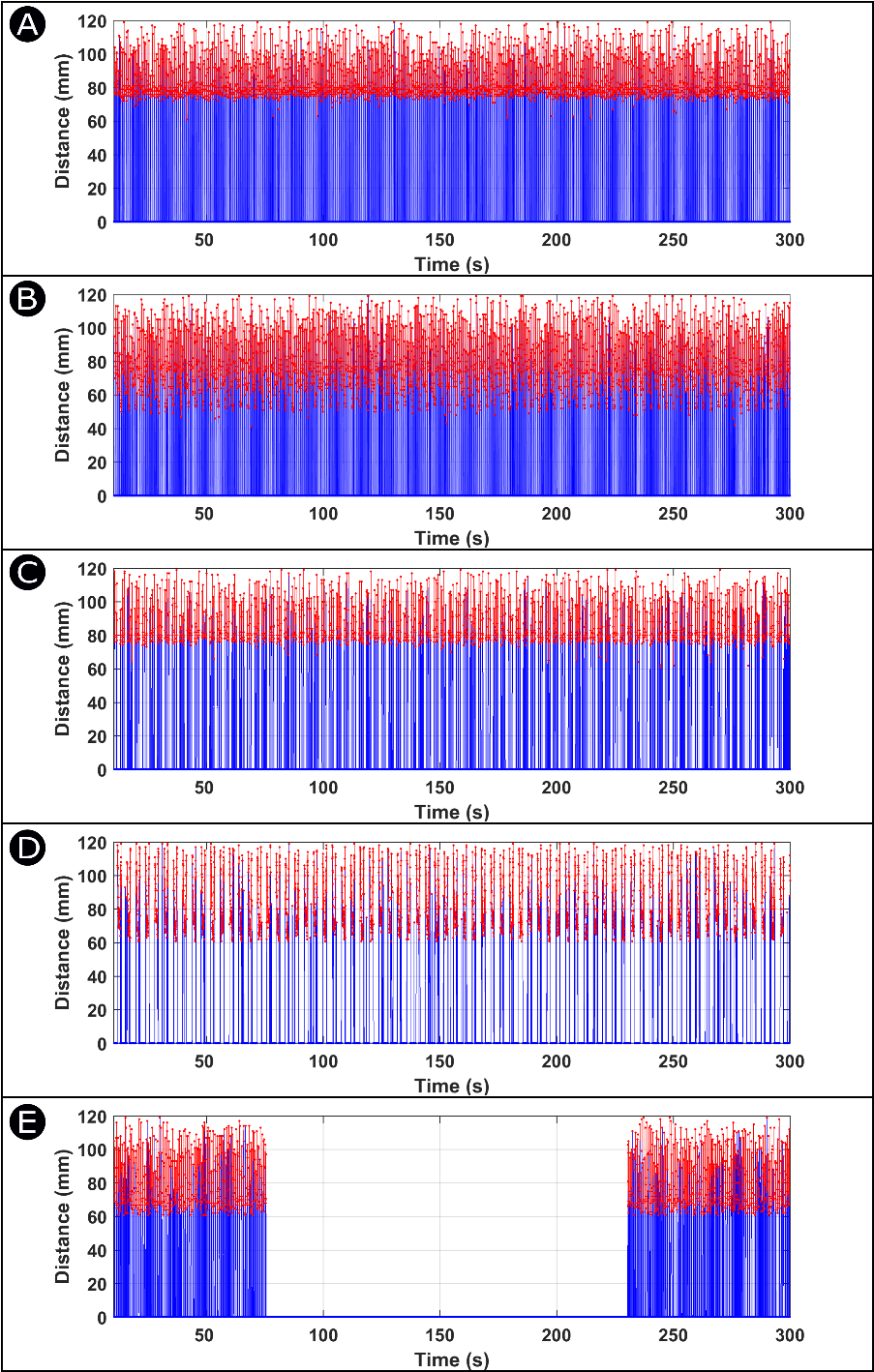


Figure S16: A figure showing the raw sensor data transmitted by the Limpet via serial communication during A) normal operation, B) Fault 1, C) Fault 2, D) Fault 3, E) Fault 4. The sensor data has been recorded over a period of 300 seconds.


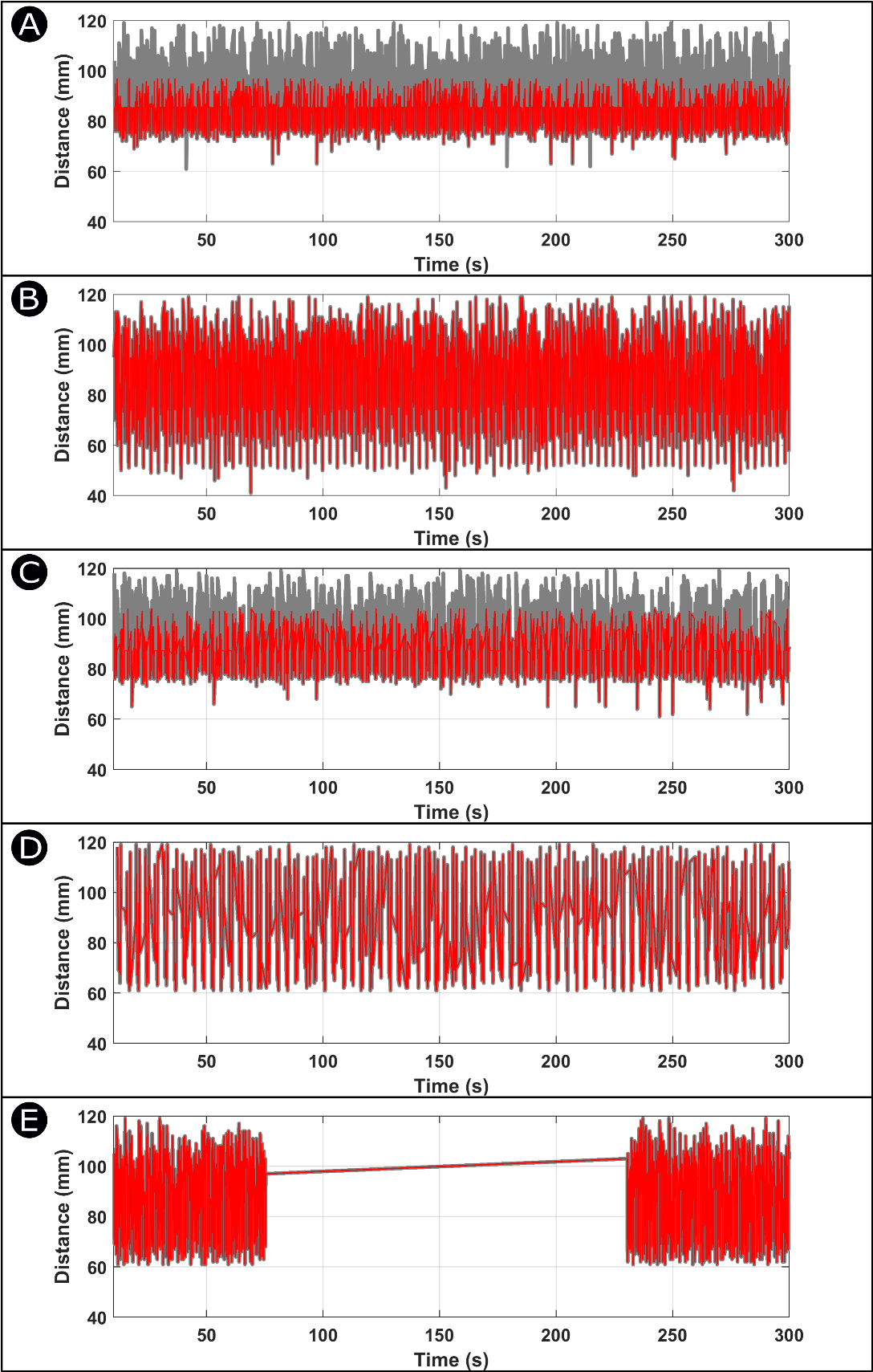


Figure S17: A figure showing the sensor data, which was transmitted via serial communication, after elimination of outliers during A) normal operation, B) Fault 1, C) Fault 2, D) Fault 3, E) Fault 4. The sensor data has been recorded over a period of 300 seconds.


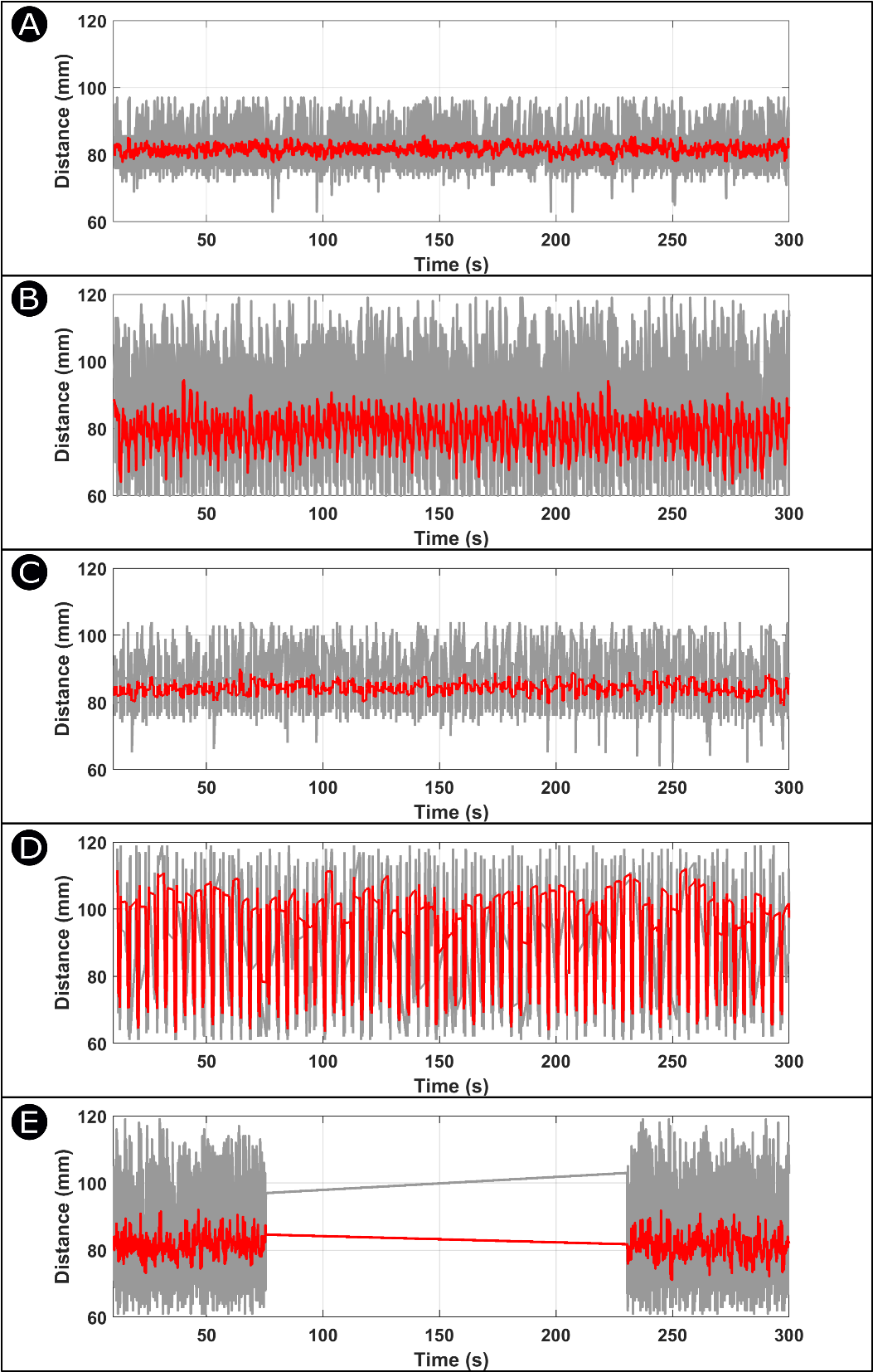


Figure S18: A figure showing the sensor data, which was transmitted via serial communication, after application of a low-pass filter during A) normal operation, B) Fault 1, C) Fault 2, D) Fault 3, E) Fault 4. The sensor data has been recorded over a period of 300 seconds.


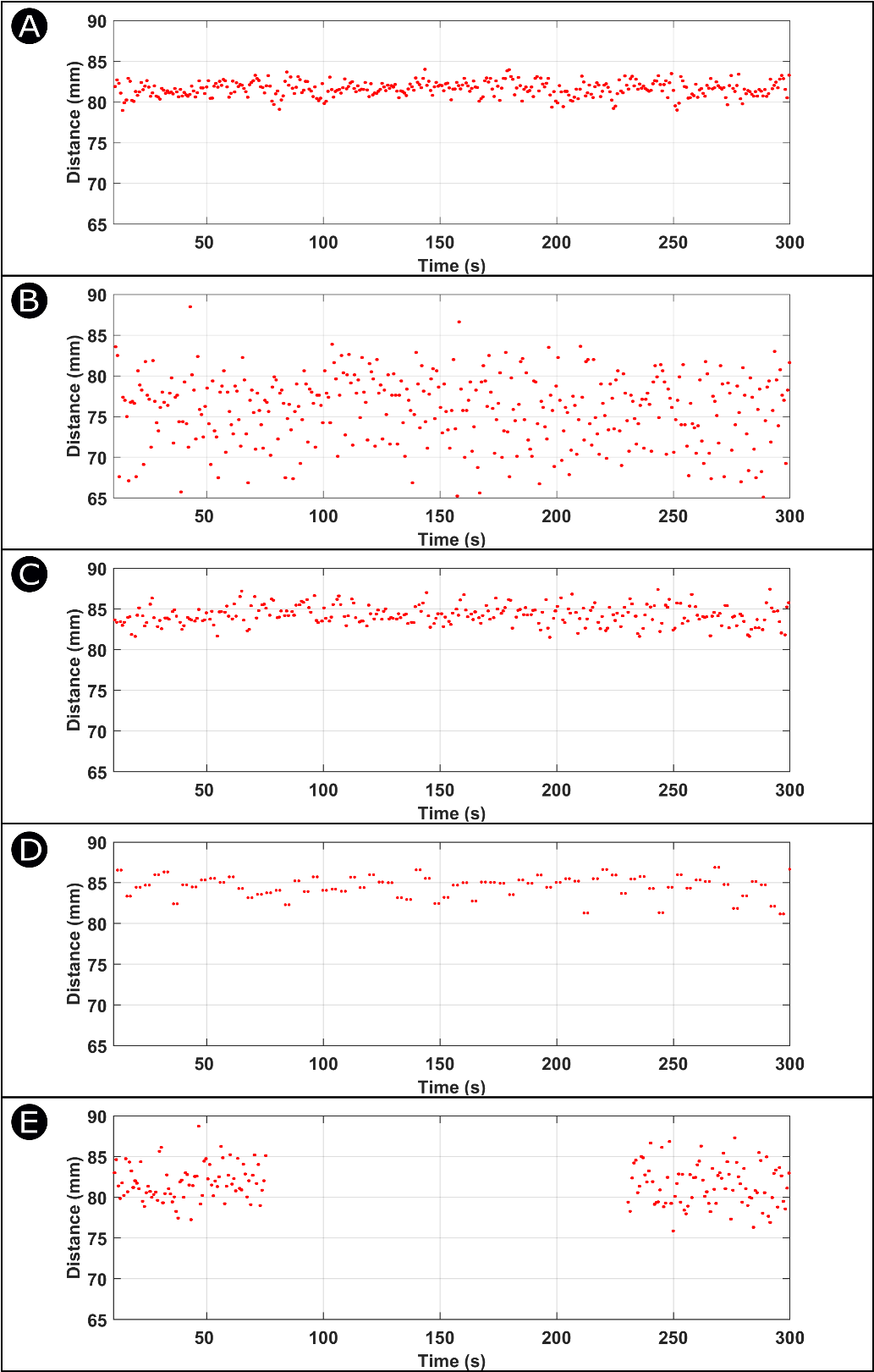


Figure S19: A figure showing the down-sampled sensor data, which was transmitted via serial communication during A) normal operation, B) Fault 1, C) Fault 2, D) Fault 3, E) Fault 4. The sensor data has been recorded over a period of 300 seconds.


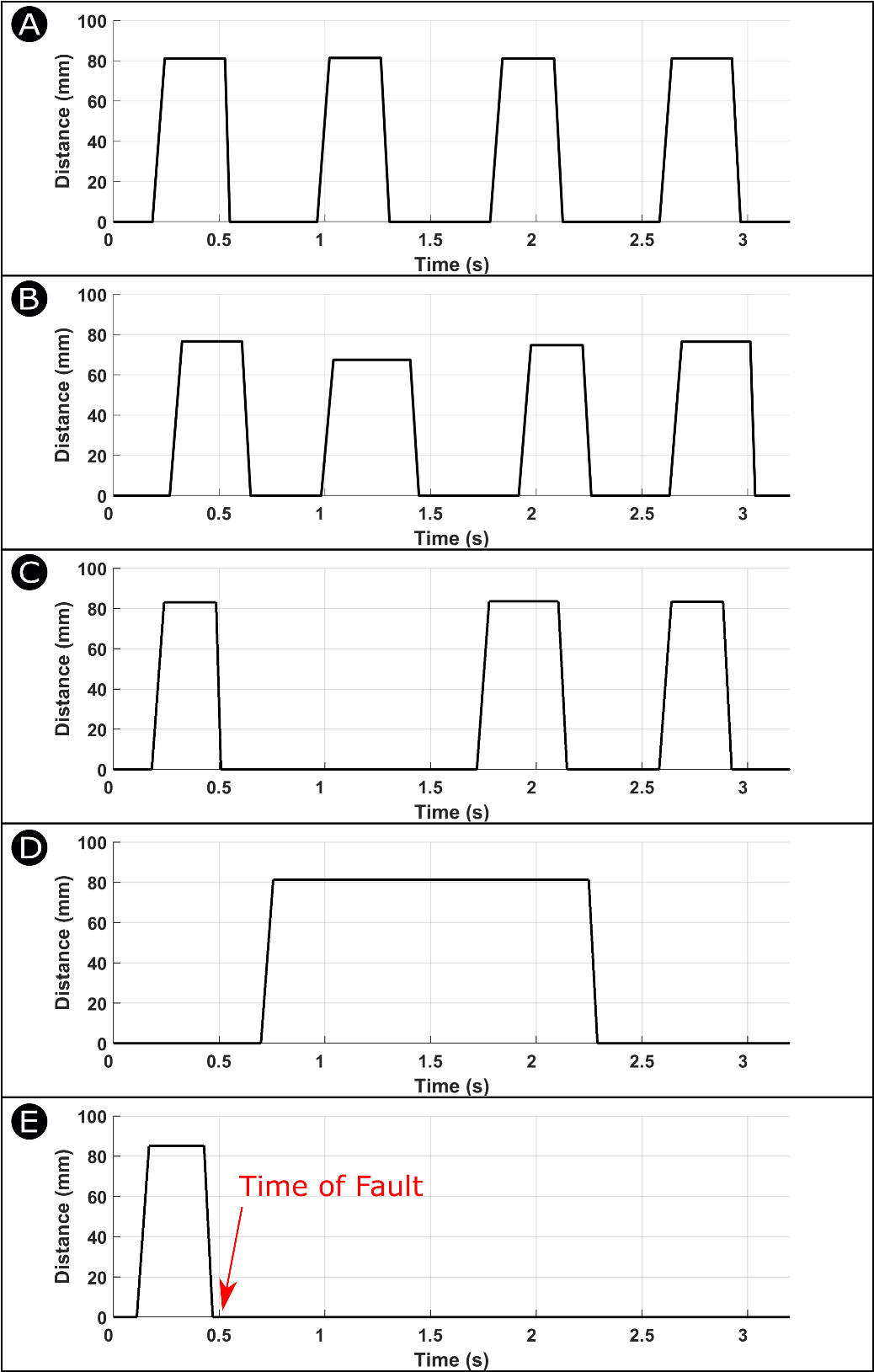


Figure S20: A figure showing the distance profile for sensor data, which was transmitted via serial communication during A) normal operation, B) Fault 1, C) Fault 2, D) Fault 3, E) Fault 4. The sensor data has been recorded over a period of 300 seconds.


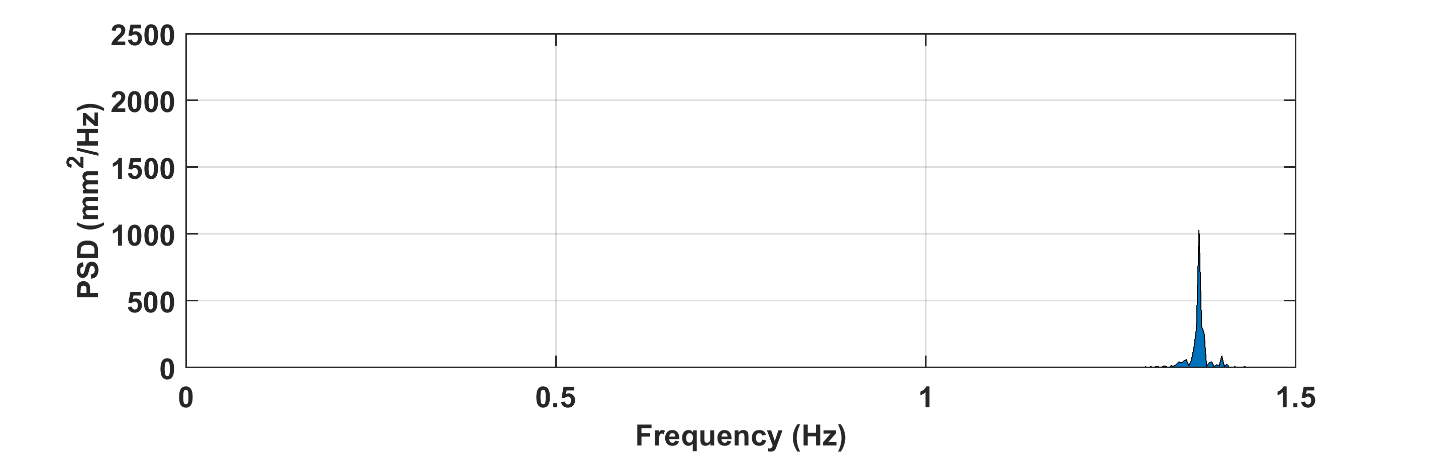


Figure S21: Power spectral density plot of normal operation mode of the fan. There is a single peak at a frequency of 1.3 Hz, which corresponds to the frequency of detection of the fan blades.


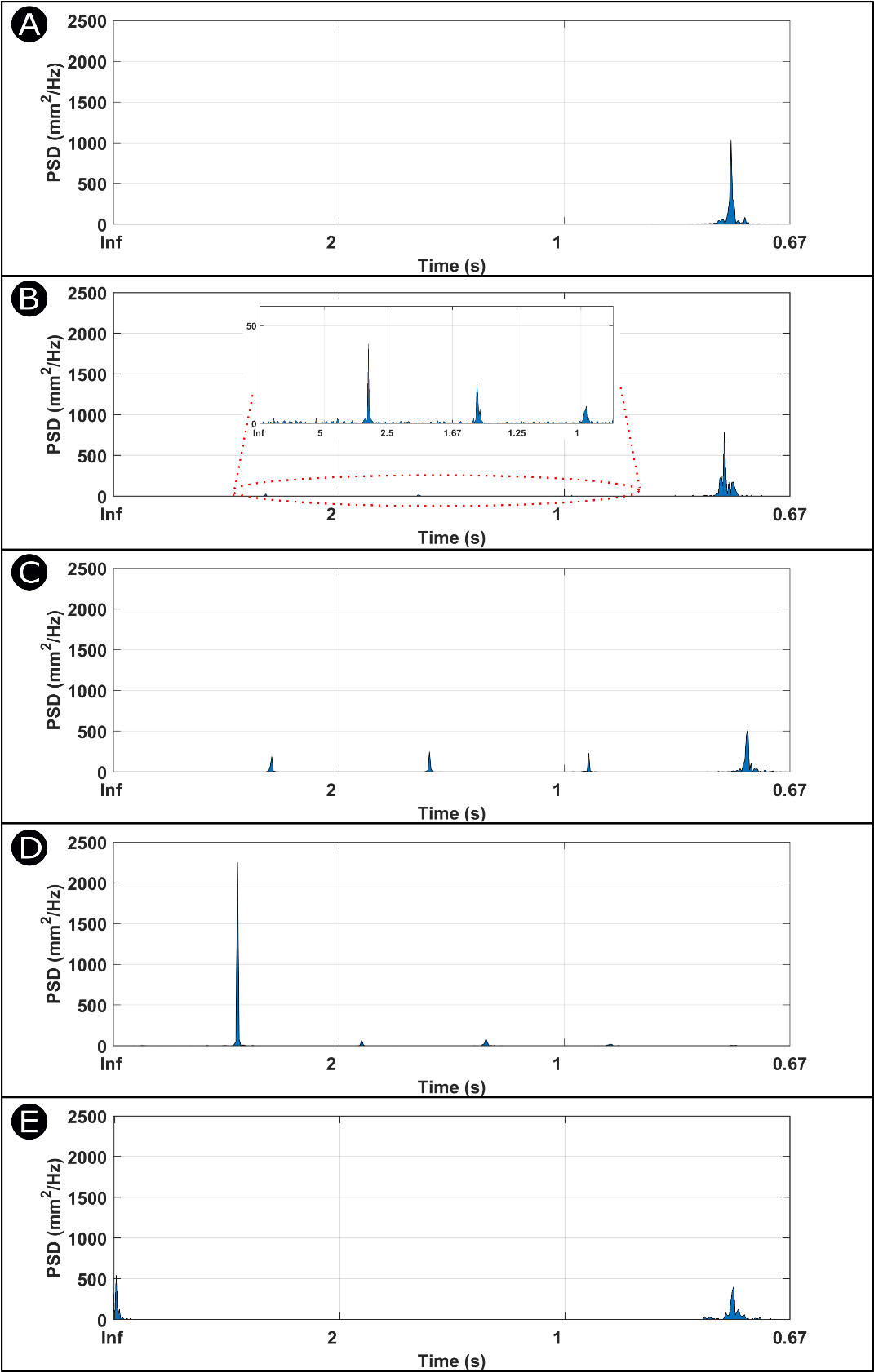


Figure S22: A figure showing the power spectral density over time applied to the sensor data that was transmitted via WiFi during A) normal operation, B) Fault 1, C) Fault 2, D) Fault 3, E) Fault 4.


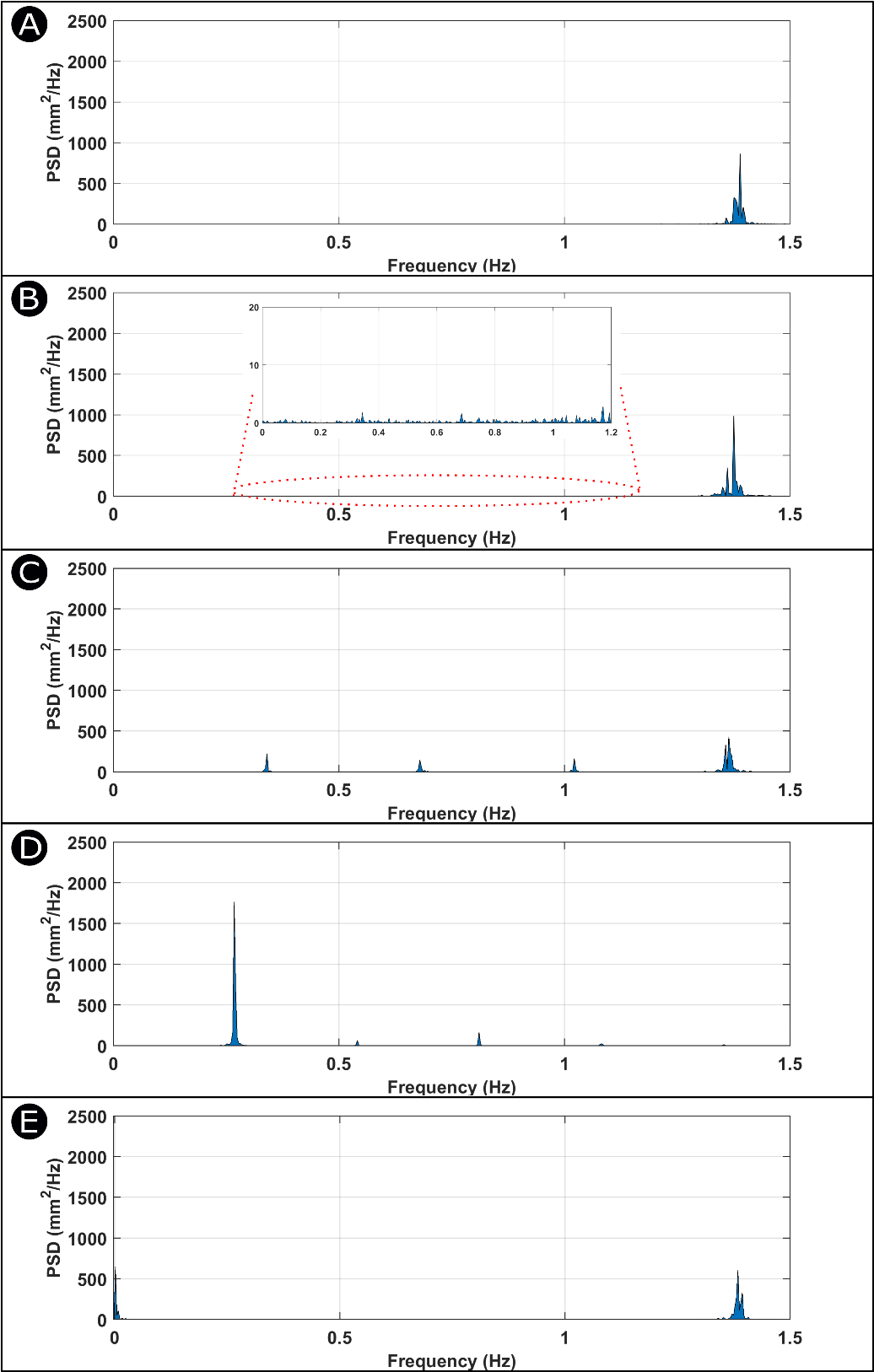


Figure S23: A figure showing the power spectral density over frequency applied to the sensor data that was transmitted via serial communication during A) normal operation, B) Fault 1, C) Fault 2, D) Fault 3, E) Fault 4.


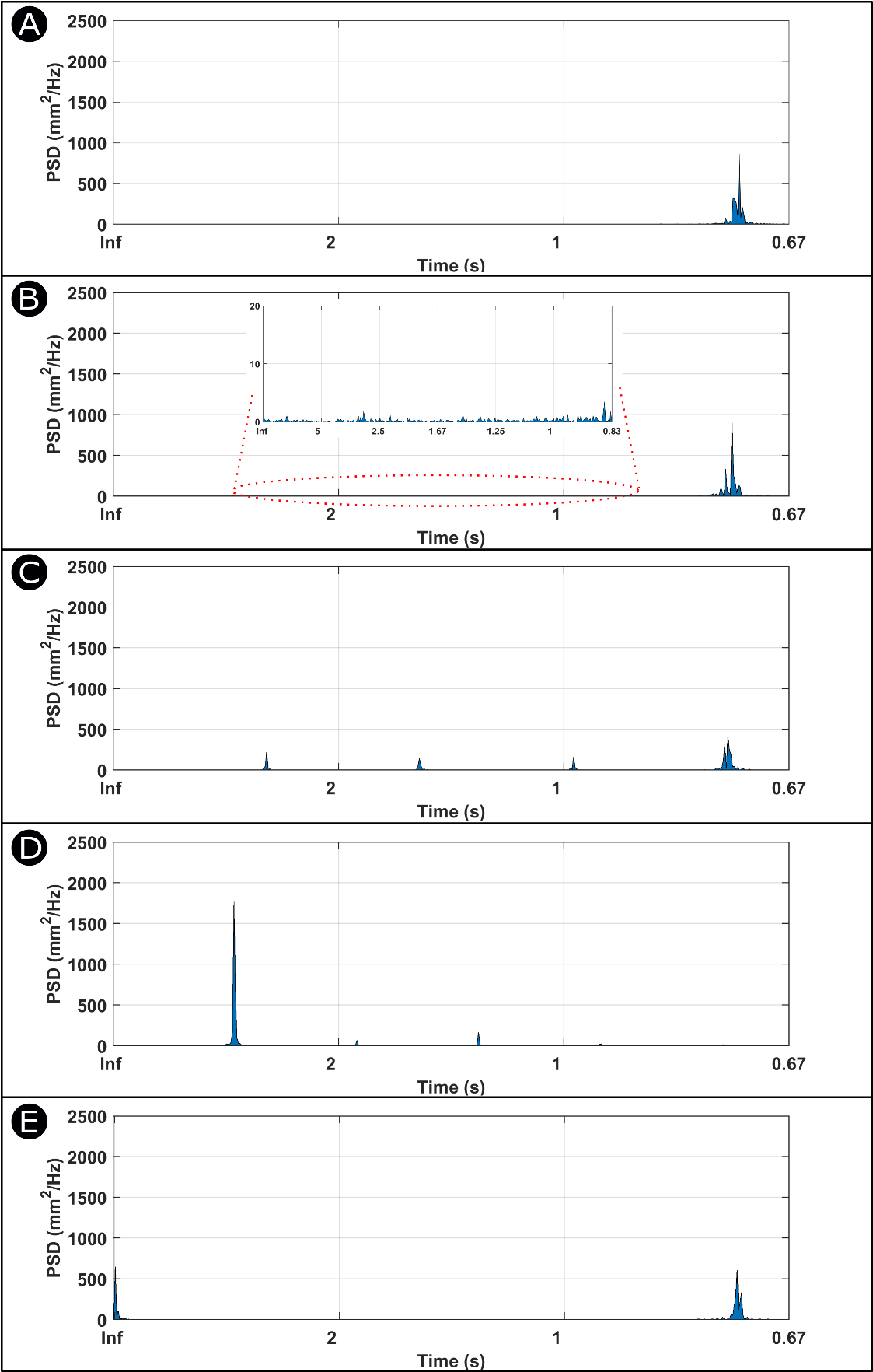


Figure S24: A figure showing the power spectral density over time applied to the sensor data that was transmitted via serial communication during A) normal operation, B) Fault 1, C) Fault 2, D) Fault 3, E) Fault 4.


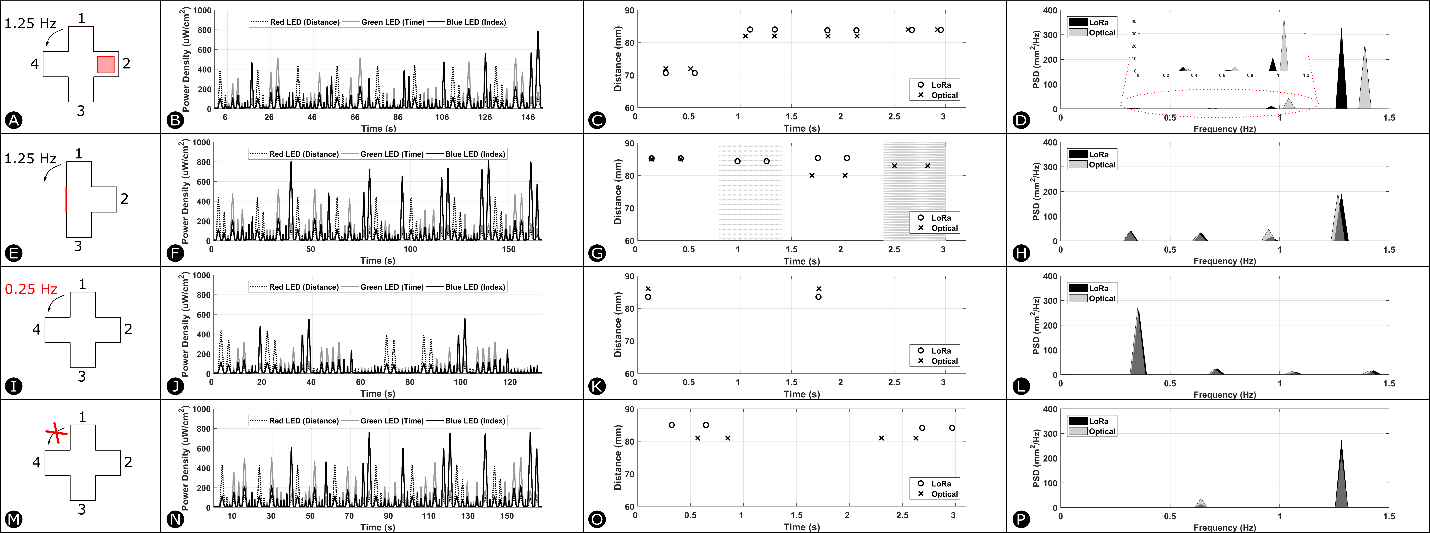


Figure S25: Results of the experiments using LoRa and optical communication. The LoRa and optical communication experiments were conducted separately. This figure shows the down-sampled results from LoRa communication and optical communication when we introduce the four faults to the system, and the spectral analysis done on the down-sampled results for each of the faults. The down-sampled results are shown within a period (T) of 3.2 seconds (corresponding to the frequency of rotation of the fan during normal operation. The figure also shows the colour power density from the optical sensor during the transmission of data. The graph contains information on the distance, timestamp and index of the measurement. The red peaks represent distance data, green peaks represent time data, and blue peaks represent index data. For each measurement, 2 distance data points are transmitted (2 red peaks), 4 timestamp data points are transmitted (4 green peaks), and 3 index data points are transmitted (3 blue peaks). A) Schematic of the first fault introduced to the system. B) This graph shows the colour power density from the optical sensor when fault 1 is introduced to the system. C) This graph shows the down-sampled result from the distance measurement, which is transmitted via LoRaWAN network and optical communication, after introducing fault 1 to the system. The optical communication results are constructed from the optical sensor data. Every two consecutive points on the graph represent a single peak (fan blade). One of the peaks in the graph has a lower value than the other peaks. D) Spectral analysis of fault 1 operation mode for LoRa and optical communication. There is a large peak around a frequency of 1.3 Hz, and three smaller peaks around frequencies of 0.35 Hz, 0.7 Hz and 1 Hz. The peaks at 1.3 Hz represents the fan blade with the attached object, while the other peaks represent the other three fan blades. E) Schematic of the second fault introduced to the system. F) This graph shows the colour power density from the optical sensor when fault 1 is introduced to the system. G) This graph shows the down-sampled result from the distance measurement, which is transmitted via LoRaWAN network and optical communication, after introducing fault 2 to the system. The optical communication results are constructed from the optical sensor data. Every two consecutive points on the graph represent a single peak (fan blade). One of the peaks in the graph is missing for each communication system. H) Spectral analysis of fault 2 operation mode for LoRa and optical communication. There are three smaller peaks at frequencies of 0.3 Hz, 0.7 Hz and 0.9 Hz representing each of the fan blades and a larger peak at 1.3 Hz representing the missing fan blade. I) Schematic of the third fault introduced to the system. J) This graph shows the colour power density from the optical sensor when fault 3 is introduced to the system. K) This graph shows the down-sampled result from the distance measurement, which is transmitted via LoRaWAN network and optical communication, after introducing fault 2 to the system. The optical communication results are constructed from the optical sensor data. Every two consecutive points on the graph represent a single peak (fan blade).The graph shows a single peak within period (T) as the fan is rotating at a lower speed. L) Spectral analysis of fault 3 operation mode for LoRa and optical communication. There is a large peak at a frequency of 0.3 Hz representing the lower frequency of rotation of the fan blades. M) Schematic of the fourth fault introduced to the system. N) This graph shows the colour power density from the optical sensor when fault 4 is introduced to the system. O) This graph shows the down-sampled result from the distance measurement, which is transmitted via LoRaWAN network and optical communication, after introducing fault 4 to the system. The optical communication results are constructed from the optical sensor data. Every two consecutive points on the graph represent a single peak (fan blade). Two of the peaks are missing corresponding to when the fault occurred. P) Spectral analysis of fault 4 operation mode. There is a peak at a frequency of 1.3 Hz representing the normal fan operation and another peak at a lower frequency representing the fault introduced to the system.


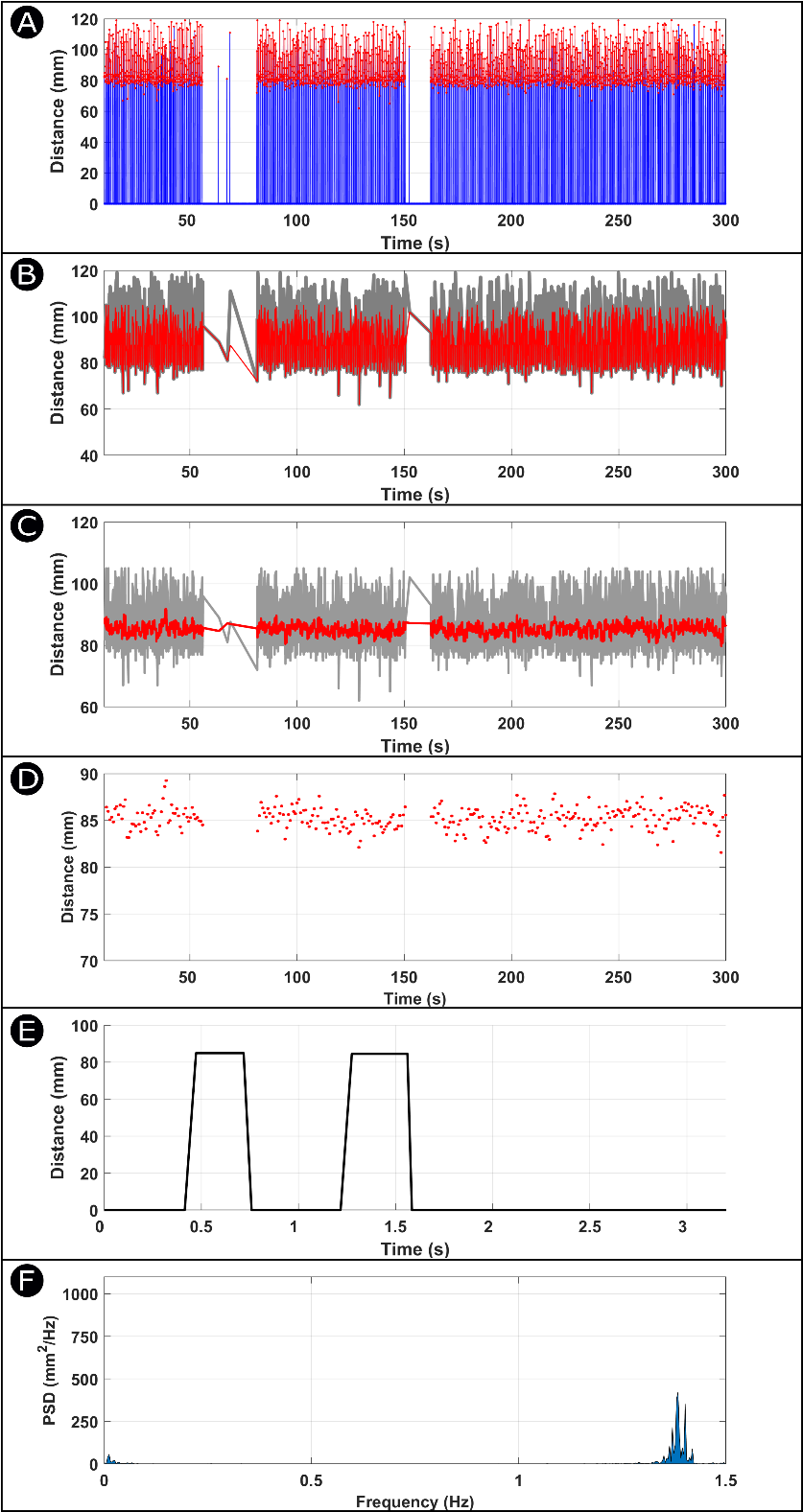


Figure S26: A figure showing a new type of fault introduced to the fan by stopping its rotation twice during operation. The sensor data was transmitted via WiFi. A) Raw sensor data. B) Sensor data after elimination of outliers. C) Sensor data after application of low-pass filter. D) Down-sampled sensor data. E) Distance profile. F) Power spectral density.


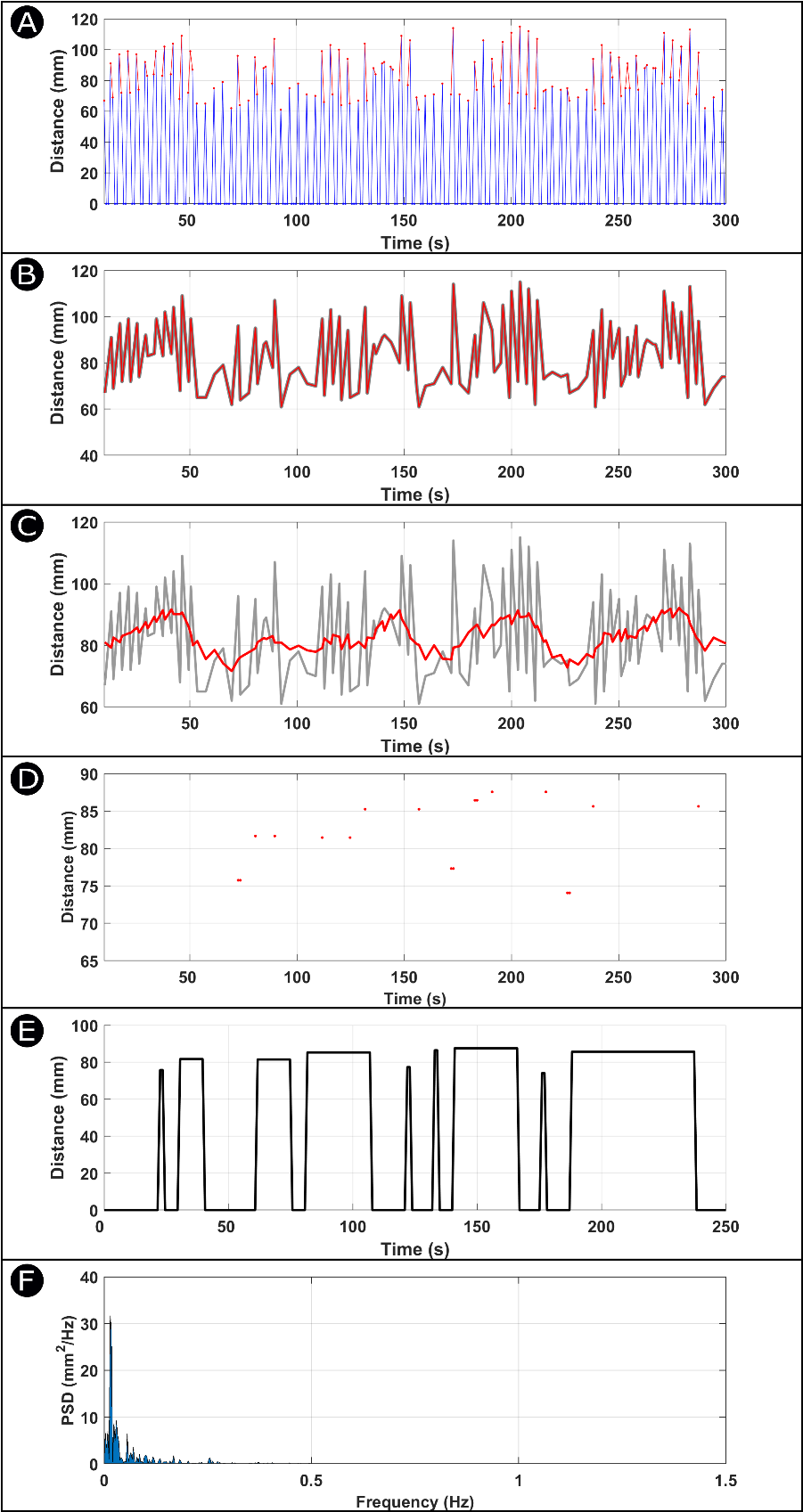


Figure S27: A figure showing an experiment conducted with a sampling rate lower than frequency of blade rotation. The sampling frequency used in this experiment is 1Hz. The frequency is lower than Nyquist frequency, showing that normal or fault measurands can not be identified. The sensor data was transmitted via WiFi. A) Raw sensor data. B) Sensor data after elimination of outliers. C) Sensor data after application of low-pass filter. D) Down-sampled sensor data. E) Distance profile. F) Power spectral density.


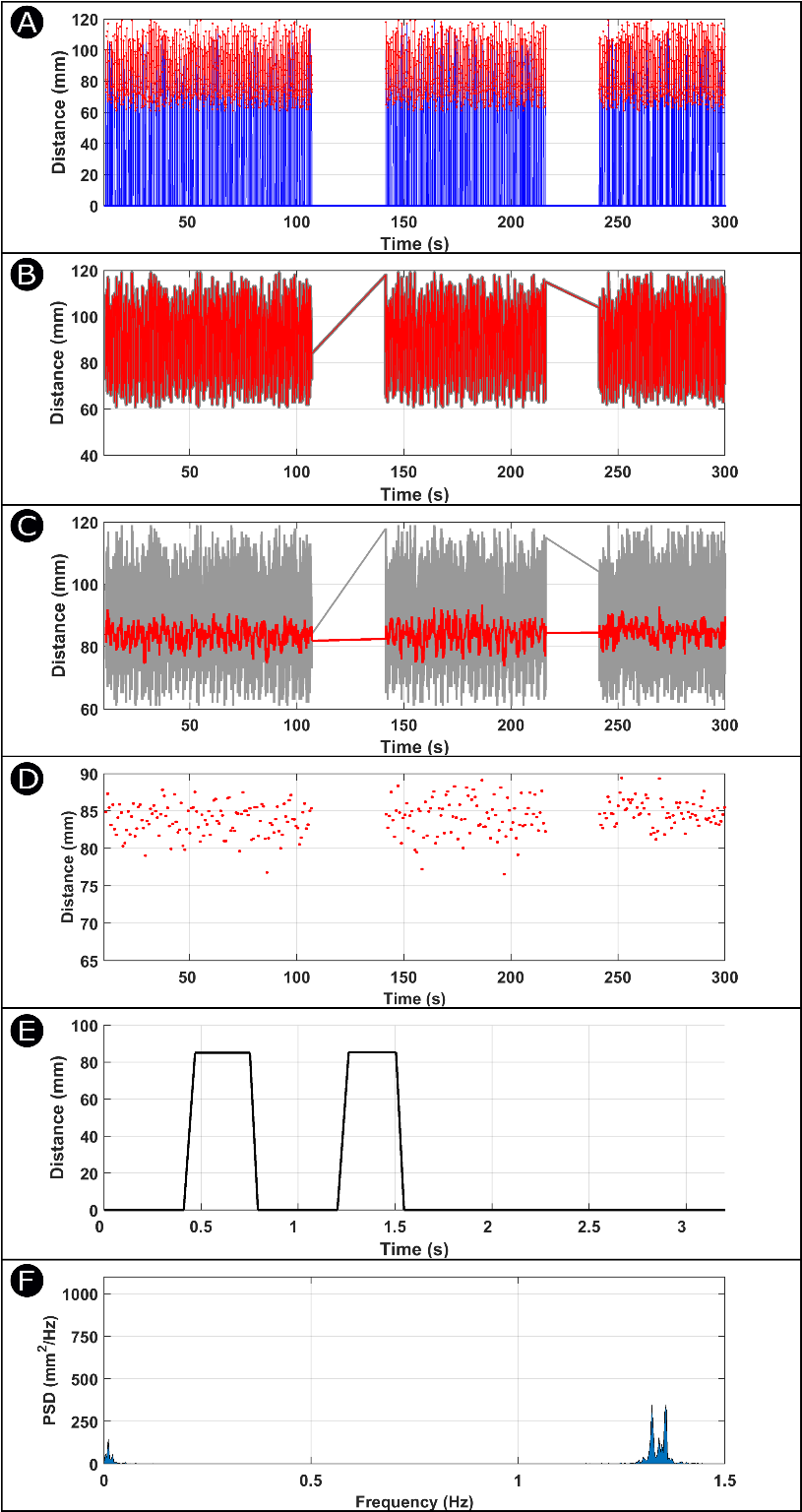


Figure S28: A figure showing a new type of fault introduced to the fan by stopping its rotation twice during operation. The sensor data was transmitted via serial communication. A) Raw sensor data. B) Sensor data after elimination of outliers. C) Sensor data after application of low-pass filter. D) Down-sampled sensor data. E) Distance profile. F) Power spectral density.


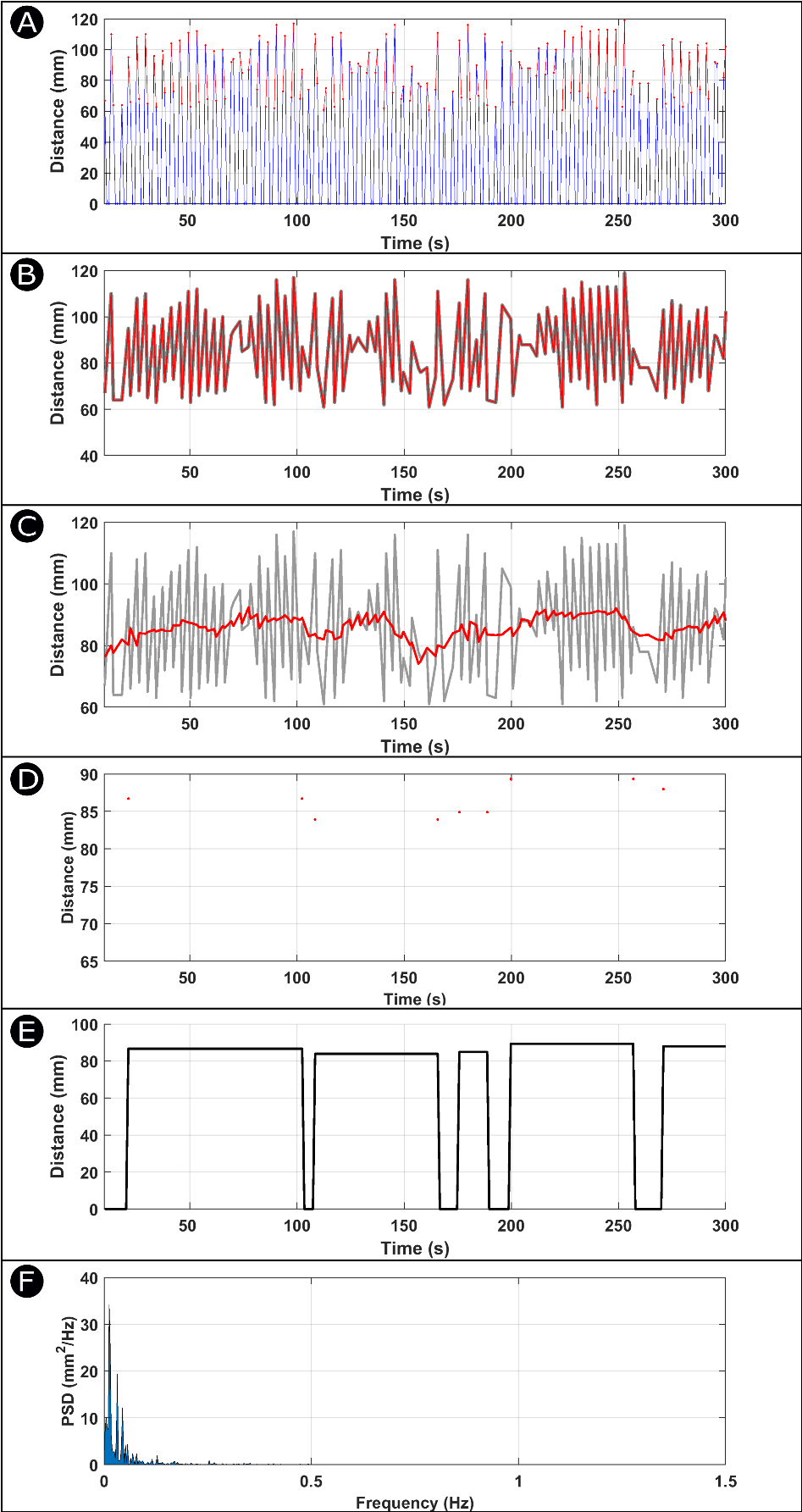


Figure S29: A figure showing an experiment conducted with a sampling rate lower than frequency of blade rotation. The sampling frequency used in this experiment is 1Hz. The frequency is lower than Nyquist frequency, showing that normal or fault measurands can not be identified. The sensor data was transmitted via serial communication. A) Raw sensor data. B) Sensor data after elimination of outliers. C) Sensor data after application of low-pass filter. D) Down-sampled sensor data. E) Distance profile. F) Power spectral density.

# Technical Files

The authors have provided a zip-file for CAD files, PCB schematics, User Guide and Bill of Materials.
